# Functional odor map heterogeneity is based on multifaceted glomerular connectivity in larval *Xenopus* olfactory bulb

**DOI:** 10.1016/j.isci.2023.107518

**Published:** 2023-08-02

**Authors:** Thomas Offner, Lukas Weiss, Daniela Daume, Anna Berk, Tim Justin Inderthal, Ivan Manzini, Thomas Hassenklöver

**Affiliations:** 1 Institute of Animal Physiology, Department of Animal Physiology and Molecular Biomedicine, Justus-Liebig-University Giessen, 35392 Giessen, Germany

**Keywords:** Nervous system anatomy, Sensory neuroscience

## Abstract

Glomeruli are the functional units of the vertebrate olfactory bulb (OB) connecting olfactory receptor neuron (ORN) axons and mitral/tufted cell (MTC) dendrites. In amphibians, these two circuit elements regularly branch and innervate multiple, spatially distinct glomeruli. Using functional multiphoton-microscopy and single-cell tracing, we investigate the impact of this wiring on glomerular module organization and odor representations on multiple levels of the *Xenopus laevis* OB network. The glomerular odor map to amino acid odorants is neither stereotypic between animals nor chemotopically organized. Among the morphologically heterogeneous group of uni- and multi-glomerular MTCs, MTCs can selectively innervate glomeruli formed by axonal branches of individual ORNs. We conclude that odor map heterogeneity is caused by the coexistence of different intermingled glomerular modules. This demonstrates that organization of the amphibian main olfactory system is not strictly based on uni-glomerular connectivity.

## Highlights

• Odor map is non-stereotypic
• Glomerular juxtaposition is not correlated to odor tuning
• Multi-dendritic MTCs project to glomeruli with broad archetypical response profiles
• Bifurcating ORN axons and multi-dendritic MTCs connect to common sets of glomeruli

## Graphical abstract

**Figure:**
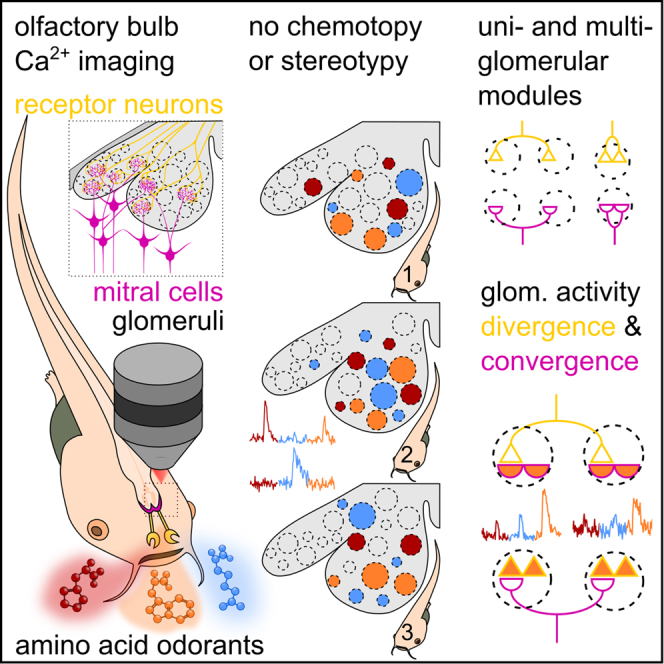

## Introduction

The array of glomeruli in the vertebrate olfactory bulb (OB) represents the first relay station of olfactory information processing. A glomerulus consists of axon terminals of receptor neurons, dendritic processes of mitral/tufted cells (MTCs), and local interneurons.^1^^,^^2^ Populations of receptor neurons expressing the same allele of a single odorant receptor gene coalesce to form distinct glomeruli.^3^^,^^4^^,^^5^^,^^6^^,^^7^^,^^8^ Odor molecule detection at the peripheral sensory surface translates to spatiotemporal glomerular activity patterns integrated by the OB neuronal network.^9^^,^^10^

At least two different modes of representing a chemosensory map on the glomerular array have been identified based on similarity of (1) odor molecule structure or (2) olfactory receptor protein. In the rodent and zebrafish main OB, glomeruli activated by structurally similar odor molecules are spatially close, and these molecular feature clusters are stereotypically arranged between animals.^11^^,^^12^^,^^13^^,^^14^ Contrastingly, in the rodent accessory OB, glomeruli with similar odor tuning are spatially dispersed.^15^ The sequence similarity of expressed receptor genes of vomeronasal receptor neurons is more indicative for the juxtaposition of connected glomeruli than molecular features of the detected odors.^15^^,^^16^ Also, glomerular input convergence is less strict, and vomeronasal receptor neurons that express a specific receptor gene project into multiple, smaller glomeruli.^7^^,^^8^^,^^17^

In a generalized olfactory system blueprint, glomerular circuit elements are primarily thought to be uni-glomerular: Individual olfactory receptor neuron (ORN) axons and mitral cell dendrites coalesce into one functionally and morphologically distinct glomerulus.^3^^,^^4^^,^^5^^,^^6^ In amphibians however, ORN axons and MTC dendrites regularly branch and innervate multiple, spatially distinct glomeruli.^18^^,^^19^^,^^20^^,^^21^^,^^22^^,^^23^

Here, we report how this wiring layout affects the functional organization of the amphibian OB. The glomerular response map to amino acid odors was neither stereotypic between individual animals nor chemotopically organized. As a potential basis for this atypical map organization in a main OB, we show that different glomerular wiring paradigms functionally overlap in the OB of *Xenopus laevis*. This highlights that the main olfactory system is not necessarily based on strictly uni-glomerular connectivity and opens up new questions about the evolutionary position of amphibians during formation of distinct olfactory wiring strategies.

## Results

We focused on the ventro-lateral OB of larval *X. laevis*, since it is best characterized in terms of signal transduction pathways, potential odorant receptor families and suitable odorants.^21^^,^^24^^,^^25^^,^^26^^,^^27^^,^^28^^,^^29^ However, none of the latter studies has investigated the spatial glomerular organization and odor map transformation by OB circuitry on both the morphological and functional level. Amino acids were shown to evoke frequent and differential glomerular responses in the lateral glomerular cluster making those stimuli a powerful choice to investigate olfactory subsystem organization.^26^

**Figure 1:**
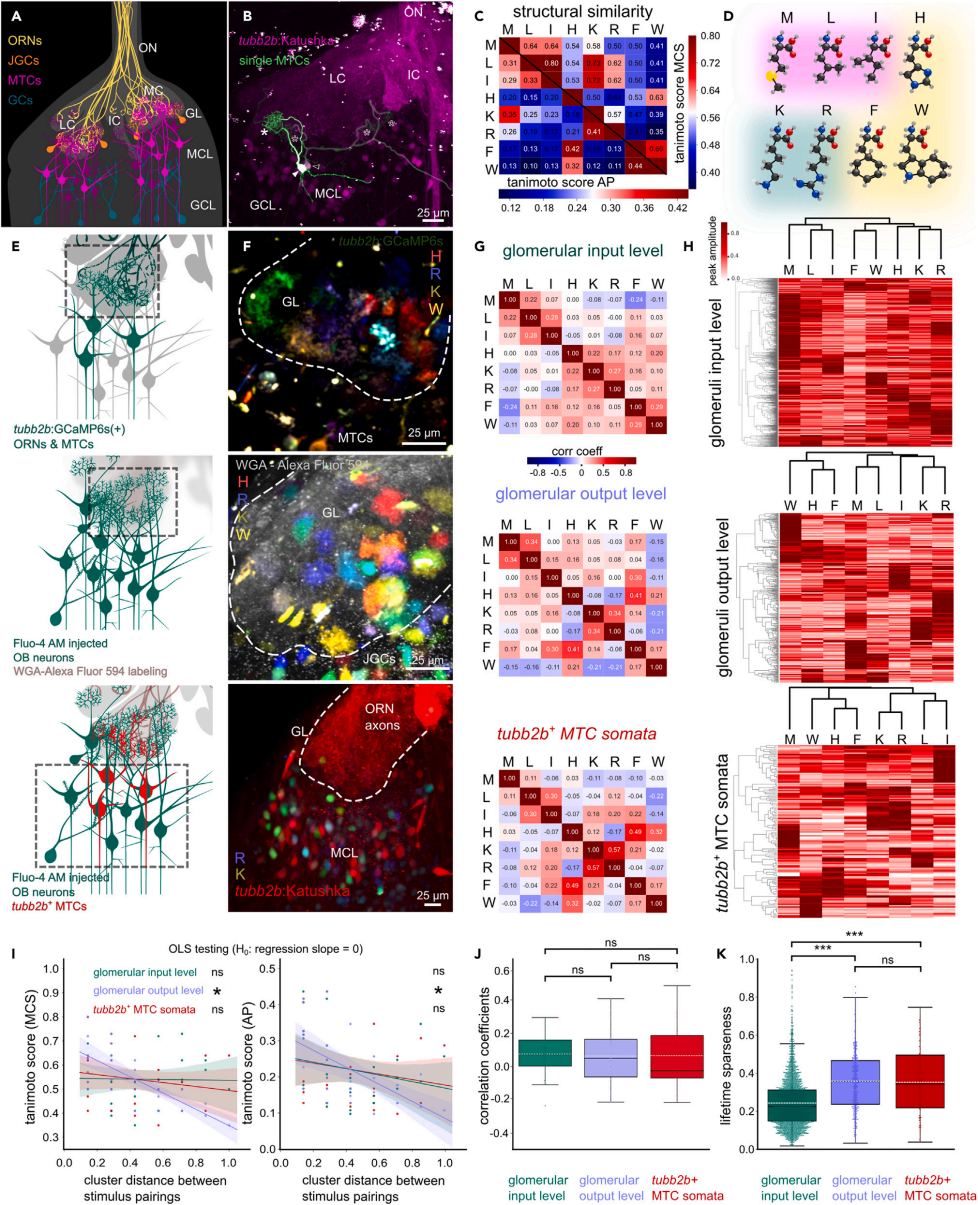
Feature selectivity to amino acids and lifetime sparseness increase from the glomerular input to the output level (A) Schematic of a main olfactory bulb (OB) hemisphere of larval *Xenopus laevis*. Olfactory receptor neuron axons (ORNs, yellow) incoming from the olfactory nerve (ON) form distinct medial (MC), intermediate (IC), and lateral (LC) clusters in the glomerular layer (GL). Dendrites of mitral/tufted cells (MTCs; magenta) and juxtaglomerular cells (JGCs, orange) make up the postsynaptic glomerular compartments. MTCs in the mitral cell layer (MCL) integrate odor information and convey it to higher brain centers after modulation by inhibitory granule cells (GCs, teal). (B) Katushka expression regulated by the *tubb2b* promoter in ORN axon terminals and a subpopulation of MTCs. Examples for uni-glomerular (filled symbols, arrowhead, soma; asterisk, dendritic tuft) and multi-glomerular *tubb2b*^*+*^ MTCs (empty symbols). Scale bar equals 25 µm. (C) Matrix of molecular Tanimoto similarity scores of amino acid stimuli (Capital letters represent single amino acid code; AP, atom pair; MCS, maximum common substructure). (D) Molecular structures of L-amino acid stimuli with structural similarity color-highlighted. (E) Experimental approaches to measure odor-induced activity on the glomerular input level (upper panel), the postsynaptic glomerular output level (middle panel), and MTC soma level (lower panel). Dashed boxes indicate area of investigation. (F) Fluorescence intensity difference maps of glomerular input (upper panel), output (middle panel), and MTC soma level activity (lower panel; H: red; K: green; R: blue; W: yellow; WGA: wheat germ agglutinin). Scale bar equals 25 µm. (G) Correlation matrices (Pearson’s correlation coefficient) of pooled response peak amplitude vectors of the glomerular input (upper panel), glomerular output (middle panel), and *tubb2b*^*+*^ MTC soma level (lower panel). (H) Hierarchical cluster analysis of response peak amplitude vectors of the glomerular input (upper panel), output (middle panel), and *tubb2b*^*+*^ MTC soma level (lower panel). (I) Normalized dendrogram column distance of stimulus pairings (cluster distance) plotted against Tanimoto similarity scores, MCS (left panel) and AP (right) for the input (green), output (blue), and *tubb2b*^*+*^ MTC level (red). Linear regressions and confidence intervals are indicated. Linear regressions between the two variables were negative and significantly different from a regression line of slope 0 (OLS testing) on the glomerular output level, only. (J) Distribution of population correlation coefficient values among the stimulus pairings (values from G). (K) Lifetime sparseness of glomerular input (green), output (blue), and *tubb2b*^*+*^ MTC soma level (red). Mean, dashed white line; median: black line; box outlines 1st and 3rd quartiles; ∗∗∗, p ≤ 0.001; ∗, p ≤ 0.05; ns, not significant, p > 0.05. See also Figures S1, S2 and S8.

We performed fast volumetric calcium imaging experiments in the lateral OB to investigate the organization and transformation of odor representations. Application of structurally diverse amino acids to the peripheral olfactory organ led to robust fluorescence changes on the level of (1) glomerular input, (2) glomerular output, and (3) MTC somata (Figures 1A and 1E). We recorded activity of glomerular input neuropil in transgenic *tubb2b*:GCaMP6s animals (Figures 1E and 1F). The *tubb2b* promoter drives expression in peripheral ORNs and a subset of MTCs^30^ (Figure 1B). Postsynaptic glomerular output responses were measured in MTC tufts and juxtaglomerular cell (JGC) neurites via Fluo-4 AM injected into the mitral cell layer (Figures 1E and 1F). Downstream somatic activity was quantified in a *tubb2b*^+^ MTC subpopulation (Figures 1E and 1F). Regions of interest with evoked calcium transients were identified in 3517 glomeruli on the input level (n = 17; *tubb2b*:GCaMP6s animals), 431 glomeruli and 299 JGCs on the output level (n = 10; Fluo-4 AM injected animals) and in 138 *tubb2b*^+^ and 387 *tubb2b*^−^ MTCs (n = 7; *tubb2b*:Katushka animals).

### Structural selectivity and contrast increase from the glomerular input to output level

Due to a specific binding pocket, odorant receptors possess a molecular receptive range which imposes a tuning profile onto ORNs expressing them.^31^^,^^32^ Differentially tuned glomeruli are associated with different odorant receptor types according to the elementary olfactory wiring principles in the vertebrate main^4^^,^^5^^,^^6^ or accessory olfactory system.^7^^,^^8^ Single amino acids have proven to be powerful odorants with both shared and highly dissimilar epitopes to study odor tuning and odor representations in the OB of aquatic animals.^14^^,^^25^^,^^26^^,^^33^ Making assumptions about (dis-)similarities in odorant receptor binding and odor tunings based on measures of molecular structure (dis-)similarity between the odor ligands is difficult. Given the shared backbone of amino acids, we chose Tanimoto scores to particularly highlight amino acid residue differences, the main contributing steric factor in olfactory receptor binding. We used a selection of eight single amino acids with different side chains and thus different physical properties (basic, aromatic, small, and long chain neutral) to decompose the OB network on multiple levels into domains of shared odor tuning, i.e., molecular feature selectivity.

To investigate the relationship between odor-evoked signals and odor molecule structure, we calculated the correlation coefficient between the pooled response peak amplitudes of stimulus pairings (Figure 1G). Amino acids were grouped by Tanimoto score similarity into (1) M, L, I; (2) K, R; and (3) H, F, and W (Figures 1C and 1D). Pairings of structurally similar amino acids R/K, W/F and M/L (Tanimoto scores AP: 0.41, 0.44. and 0.31; Tanimoto scores MCS: 0.57, 0.69, and 0.64) showed weak positive correlation on glomerular input (0.27, 0.29, and 0.22) and output (0.34, 0.17, and 0.34) level (Figure 1G). Responses to structurally dissimilar amino acids R/W and K/H (Figures 1C and 1D) had a weak positive correlation on the glomerular input level (0.11 and 0.22) and weak negative correlation on the glomerular output (−0.21 and −0.08) level (Figure 1G). Hierarchical cluster analysis of response peak amplitude vectors on the glomerular input and output level revealed tight linkage between M, L, and I, K and R, and H, F, and W. Notably, on the glomerular input level, H is linked closer to K and R. This linkage was also apparent on the *tubb2b*^+^ MTC level except for M clustering at a higher distance to L and I (Figure 1H).

We next assessed if clustering of glomeruli according to response profile correlation was related to molecular similarity scores. A negative relation between both measures would imply that glomerular clustering according to activity similarity (low cluster distance) is correlated to the structural similarity of the stimulus pairings (Tanimoto scores). We found a significantly negative trend relation between cluster distances of stimulus pairings and the respective Tanimoto MCS and AP scores on the glomerular output level (Figure 1I; OLS testing, H_0_: regression slope = 0). Higher structural similarities between stimulus pairings were connected to lower cluster distances. While the overall distributions of correlation coefficients between the pooled stimulus-response amplitudes showed no significant difference, the median correlation coefficient of the *tubb2b*^+^ MTC soma level was more negative than of glomerular input and output level (Figure 1J). The range of correlation coefficient values gradually increased on each level indicating enhanced capacity to show contrasting activity to structurally different odor molecules (Figure 1J).

To characterize odor tuning across the three OB levels in a more general, stimulus-independent manner, we calculated the lifetime sparseness of each glomerulus/MTC (see STAR methods). A larger fraction of glomeruli of the output level exhibited higher lifetime sparseness values and mean lifetime sparseness of the glomerular output/MTC soma level were significantly increased compared to the glomerular input level (Figure 1K). Mean lifetime sparseness between glomeruli of the output and *tubb2b*^+^ MTC soma were not significantly different (Figure 1K). The distribution of averaged response amplitude differences between different amino acid pairings also supported our findings (Figure S2). Altogether, we observed an increase in structural and overall tuning selectivity from glomerular input to the output level. While the lifetime sparseness of MTC somata was comparable to the glomeruli of the output level, the negative relation between response profile similarity and molecular similarity of the stimuli was not conserved.

As a next step, we wanted to understand whether individual or groups of similarly tuned glomeruli occurred in all animals and whether they were comparable in number and distribution between animals.

### Glomerular regions are dominantly tuned to one or a small set of structurally related amino acids

**Figure 2:**
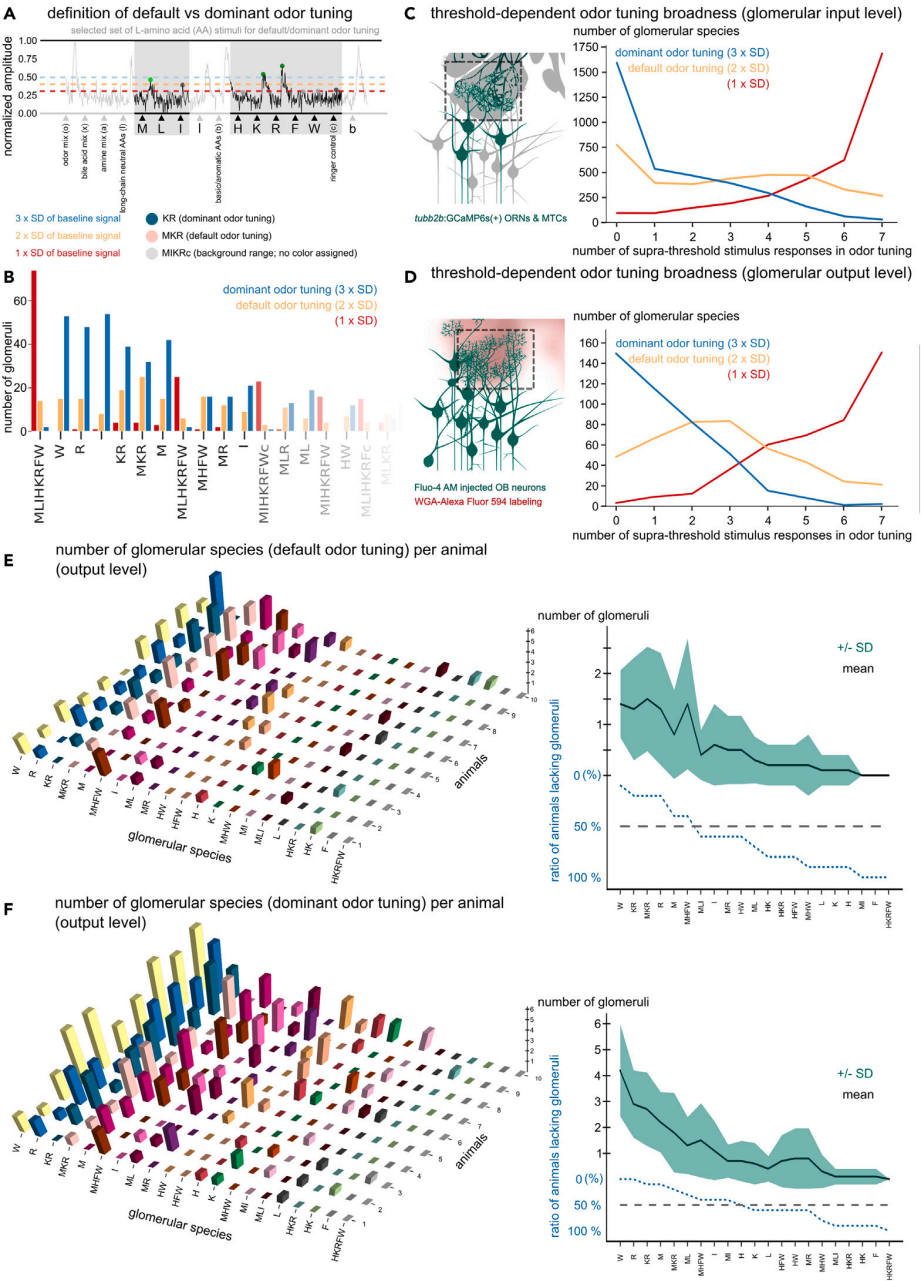
Glomeruli are dominantly tuned to a small set of structurally similar amino acid odors and are variably present in individual animals (A) Classification of odor tuning by amplitude thresholds. Calcium transients were normalized to the maximum amplitude of the experiment (in this case odorant mix: o). Responses to selected stimuli (gray rectangles) were considered, when the maximum amplitude in the stimulation interval (dots) exceeded twice the standard deviation of baseline fluorescence signal (all green dots, default odor tuning). Responses larger than triple the standard deviation were considered dominant (dark green dots, dominant odor tuning). Default/dominant odor tunings are written as sequence of the respective single amino acid code letters of all selected supra-threshold responses. (B) Frequency distributions of individual glomerular species are dependent on classification threshold. (C and D) Response-threshold dependent odor tuning broadness (number of selected stimuli causing supra-threshold responses) on the glomerular input and output level. (E and F) Presence of glomeruli with similar tuning in different individuals. 3D bar plots of glomerular species frequencies (left panels). Mean number of glomerular species ranked according to their occurrence in each animal (right panels). Many tuning profiles were not found in every individual (blue dotted line: ratio of animals lacking glomeruli). SD, standard deviation. Amino acid stimuli are represented with their capital, single letter code, mixtures of amino acids or other odorants with lowercase letters (b: basic-aromatic, s: short-chain neutral, l: long chain neutral amino acid mixtures; x: bile acid, a: amine; o: all odorants mixtures; c: frog ringer control). See also Figure S3.

Odor responses were complex, with graduated amplitude differences between amino acid stimuli. To identify preferred activation by specific odors, we classified responsive regions by amplitude thresholding (Figures 2A and 2B). We chose two different thresholds to correctly represent that amino acid response profiles typically exhibited dominant response peaks to one or few amino acids in addition to more variable, lower amplitude responses (Figures 2C and 2D). With increasing amplitude threshold, the number of regions tuned to one or few amino acid stimuli increased on both the glomerular input (Figure 2C) and output level (Figure 2D).

### Identity and frequency of glomerular regions varies between specimen

The identity and frequency of glomeruli with similar default or dominant odor tuning (glomerular species; Figures 2E and 2F) varied strongly between individual animals. While certain glomerular species (W, R, KR, M) were present in the majority of animals on the glomerular output level, others were not found in more than half of the animals (Figures 2E and 2F). Depending on tuning classification, we found an average of 1–4 ± 1–2 glomeruli with similar tuning (Figures 2E and 2F; right panels). Lack of stereotypical glomerular numbers between animals was also observable on the glomerular input level (Figures S3A and S3B). Even though glomerular species were variable in numbers in our findings, we wanted to test whether any of them showed positional consistency among the glomerular array.

**Figure 3:**
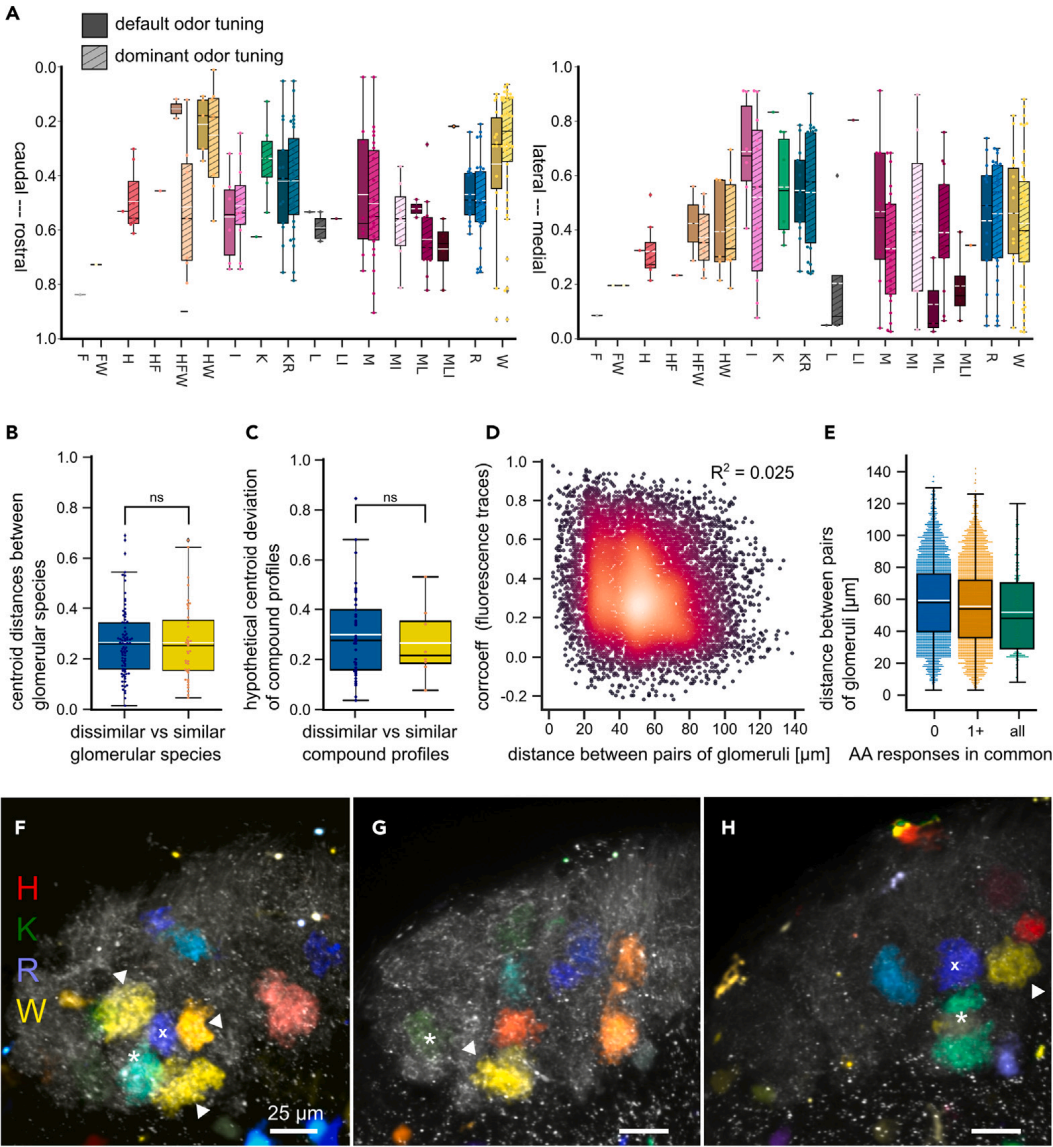
Glomeruli in the lateral cluster are positioned non-chemotopically, but individual glomerular species feature a positional bias (A) Boxplots of relative positions of glomerular species along the caudo-rostral (left panel) and medio-lateral axis (right panel) of the lateral olfactory bulb. Shading of boxes indicates odor tuning (darker shades) and dominant odor tuning (lighter shades, striped). (B) Comparison of inter-centroid distances between paired glomerular species of similar (yellow, combinations of H, F and W, K, R or M, L, I) and dissimilar (blue) amino acids. (C) Positional deviation of glomerular species with compound profiles (dominant odor tuning to two or three stimuli, e.g., HFW) from the hypothetical centroid of glomerular species tuned to corresponding single amino acids only (e.g., H, F, and W). Comparison between positional deviation from the centroid of glomeruli with compound profiles to structurally similar amino acids (yellow) and glomerular species of all remaining combinations of compound profiles (blue). (D) Interglomerular distance plotted against the correlation coefficient between time traces of all glomerular pairings (density indicated by color gradient purple to orange; n = 10). Lack of linear positive or negative relation (R ^2^ = 0.025) confirmed by OLS testing H_0_: regression slope = 0). (E) Comparison of distance between glomeruli based on common tuning (blue, no common stimulus; orange, at least one common stimulus; green, all stimuli in common). Mean, dashed white line; median: black line; box outlines 1st and 3rd quartiles; ns, not significant, p > 0.05. (F–H) Examples of variable glomerular organization in different individuals. Sections of the lateral glomerular cluster with intensity difference map-based representations of glomerular output level activity to the amino acids H, K, R, and W (red, green, blue, yellow). The juxtaposition of glomeruli responsive to W (white arrowheads) and glomeruli responsive to structurally dissimilar amino acids R (white cross) or K (white asterisk) is highlighted. All glomeruli were labeled with wheat germ agglutinin (gray). Scale bar equals 25 µm. See also Figures S4–S6.

We investigated the location of glomerular species within the lateral cluster along the medio-lateral, caudo-rostral and ventro-dorsal axis (Figure 3). We found glomerular species (e.g., W or HW) with a strong caudal bias and an intermediate position along the medio-lateral axis (Figure 3A). Glomerular species tuned to I were slightly biased toward the lateral part of the glomerular cluster, but spread along the medio-lateral axis (Figure 3A). Overall, we observed high positioning variability between glomerular species independent of odor tuning classification (Figures S4A–S4F).

### Organization of glomeruli is not chemotopic and juxtaposition does not indicate odor tuning similarity

Coarse chemotopical maps and molecular feature clusters have been described especially on the macroscopic level of the OB.^11^^,^^12^^,^^13^^,^^25^ However, there is also the notion of fractured domains of glomeruli mapped according to tuning similarity, chemical groups or structural features on the microscopic glomerular level.^14^^,^^34^^,^^35^^,^^36^ We wanted to find general consistencies and principles in arrangement of glomeruli among the lateral cluster related to their tuning.

To check for organization according to a molecular receptive range, we analyzed spatial territories of glomerular species on the output level dominantly tuned to structurally similar amino acids (Figures 3A, S4G, and S4H). The territories of those individual groups were largely overlapping. We found no differences in distances between centroids of glomerular species tuned to similar (three groups: H, F and W; R and K; M, L and I) vs. dissimilar (all other stimulus pairings) amino acids (Figure 3B). To assess the possibility of gradual, structure-based transitions of odor representations across the glomerular cluster, we analyzed whether the centroids of glomerular species with compound profiles (e.g., HFW) were located near the hypothetical centroid defined by the glomerular species tuned to the single stimuli only (e.g., H, F, and W). We did not find significant differences in the hypothetical centroid deviation (Figure 3C), indicating that trajectories across the glomerular cluster do not imply gradual changes of odor representations between reference points.

Next, we explored how glomerular tuning was related to that of other adjacent glomeruli. We found no linear relation (OLS regression analysis, R^2^ = 0.025) between the inter-glomerular Euclidean distance and the correlation coefficient between raw fluorescence traces of glomerular pairings (Figure 3D). Also, the number of common stimuli in dominant odor tunings of glomeruli showed no obvious dependence on inter-glomerular distance (Figure 3E). We frequently found glomeruli tuned to structurally dissimilar amino acids (e.g., W vs. R or K) close to each other in multiple animals (Figures 3F–3H). In conclusion, juxtaposition of glomeruli was a poor predictor of similarity in odor tuning.

### Bifurcating ORN axons can be associated with amino acid-responsive glomeruli

**Figure 4:**
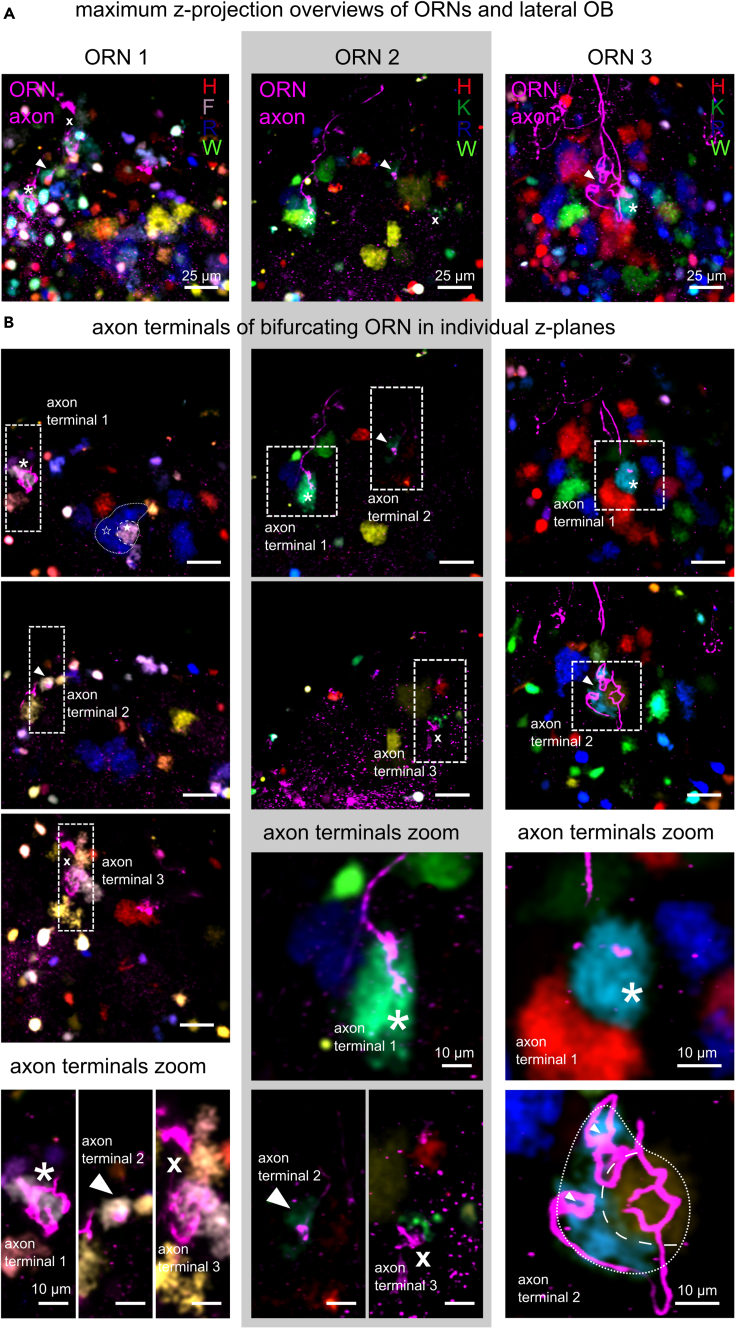
Axonal terminals of multi-glomerular ORNs can be associated with similarly tuned glomeruli (A) Maximum intensity projection of the lateral olfactory bulb showing three ORN axons (magenta) with terminals (arrowhead, asterisk, cross) connected to postsynaptic glomerular neuropil (color-coded by responsiveness to single amino acids, H, red; K, green; R, blue; W, yellow; F, gray). (B) Individual imaging planes of the surveyed volume with their axon terminals (arrowhead, asterisk, or cross) and associated glomeruli highlighted. Scale bar equals 25 µm. Lower panels show a higher magnification of axon terminals (dashed white rectangles). Scale bar equals 10 µm.

As a next step, we examined how a seemingly inconsistent glomerular organization is connected to the wiring of the OB inputs. In amphibians, ORN axons frequently bifurcate to innervate distinct glomerular targets. We labeled individual ORNs and recorded amino acid-evoked responses to determine whether glomerular target regions innervated by the same ORN are tuned to amino acids (Figure 4). We labeled 18 multi-glomerular ORNs and found six to be associated with glomerular regions responsive to amino acids. Labeled ORNs were variable in axon terminal segregation, complexity, and size. Four glomeruli innervated by single ORNs were associated with basic amino acids K and R, but also glomerular reactivity to aromatic amino acids as F and W occurred. While in our experiments, axon endings of multi-glomerular ORNs projected to similarly tuned glomeruli, the sample size did not allow conclusions about general properties of glomeruli innervated by multi-glomerular ORNs. Especially, since a fraction of them seemed to be amino acid insensitive. In one ORN, we found axon terminals branching into bipartite glomerular regions with differential tuning to amino acids (Figure 4, ORN3). We could not exclude a functional synaptic connection into these regions. This supports a divergence of olfactory information into glomeruli with the same or different odor tuning.

### MTC innervate distinct glomerular units in a coarse topological manner

This raises the question of how the postsynaptic glomerular network is organized to process a divergence of odor input. We assessed this first on the population level, by comparing the distribution of glomerular species on the output level to the positioning of MTC somata we recorded in *tubb2b*:Katushka animals according to their dominant odor tunings. Similar to the glomerular output level (Figure S5A), *tubb2b*^+^ MTCs tuned to W or HW showed a caudal bias at an intermediate position along the medio-lateral axis (Figure S5B). MTCs tuned to I were biased toward a lateral position (Figure S5B). While a coarse topological bias of individual glomerular species in regard to MTC somata of similar tuning seemed to be present, *tubb2b*^*+*^ MTCs represented only a fraction of the MTC population.

We thus investigated the dendritic projection pattern of MTCs by local labeling of MTC subpopulations in the lateral OB via fluorescent dye injection (Figure S1D). Distinct glomerular units of the unparcellated lateral glomerular cluster (Figure S5C) were innervated by similarly sized, tufted dendritic projections of MTCs of different somatic positions (Figures S5C–S5I). Lateral, intermediate, or medial dye injections into the mitral cell layer labeled laterally (Figure S5G), intermediately (Figure S5H) or medially (Figure S5I) biased dendritic projection fields among the cluster, respectively. We analyzed the spatial distribution of pixels in the glomerular and MTC layer that showed at least a 2:1 ratio of color intensity to the other respective dyes (classified by the dominant color into GL or MCL blue, green and red color category). In all five animals analyzed, we saw a clear topological link of MTC somata and the glomeruli of similar color category (Figure S6, two animals displayed: A–D and E–H). Overall, we observed a topological arrangement of glomerular modules in the lateral OB despite the lack of chemotopy, stereotypy, and consistency in glomerular wiring.

### MTCs are classified by the number of dendritic tufts

**Figure 5:**
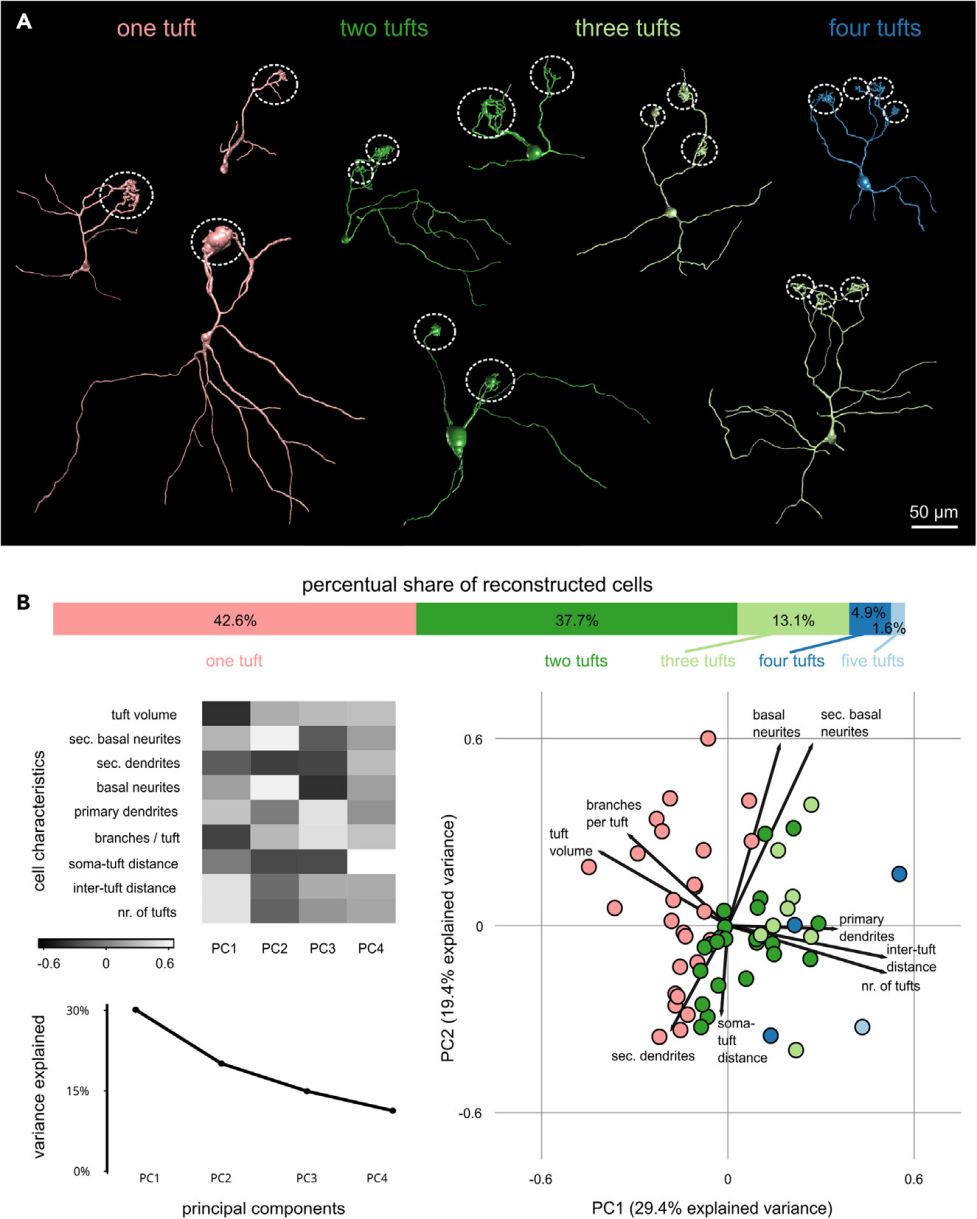
MTCs are morphologically heterogeneous, mainly classifiable by their glomerular wiring strategy (A) Representative reconstructions of labeled MTCs show variability in number of dendritic tufts (white dotted circles). Uni- and bitufted cells have the highest percentage share (pink, dark green), while multiple tufts are rarer. Scale bar equals 50 µm. (B) Principal component analysis (PCA) of nine morphological MTC characteristics. Heatmap of correlations of individual parameters with principal components (PC, gray; upper and lower left panel). Mapping of MTCs (circles) in PC1 and PC2 space. Colors represent the number of dendritic tufts (right panel). Eigenvectors are indicated as arrows.

After investigating MTCs of the lateral cluster on the population level, we aimed to dissect the MTC population further in terms of morphological and functional subtypes contributing to the glomerular module circuitry. We reconstructed the morphology of MTCs of the lateral OB labeled via single/sparse cell electroporation (n = 61, Figure 5A). MTCs with single (42.6%) or two (37.7%) dendritic tufts were most common, while three (13.1%), four (4.9%) and five (1.6%) tufts were considerably less abundant. The analyzed MTC population was generally heterogeneous and clear subgroups could not be detected based on morphological features using principal component analysis (PCA, Figure 5B). Principal component 1 (PC1) accounts for almost 30% of the variance and approximately mirrors the variation between uni- and multi-tufted cells. The analysis suggests a trade-off between the number of tufts and the number of primary dendritic stems from the soma (positive correlation with PC1) and the tuft volume and the number of dendritic branches entering each tuft (negative correlation with PC1). Tufts of uni-tufted MTCs had an average volume of 3118 ± 2970 μm³ (n = 26 tufts), while bitufted MTCs had 1655 ± 1863 μm³ (n = 46 tufts), and tufts of multi-tufted MTCs had 605 ± 812 μm³ (n = 41 tufts). In terms of number of secondary dendrites and primary/secondary basal neurites, the population was very heterogeneous, and we found no correlation between the number of tufts and the number of neurites. Number of primary and secondary basal neurites across the population averaged around 1.6 ± 1.2 and 3.4 ± 2.8, respectively. However, the number of basal neurites and secondary dendrites were anticorrelated.

### Multi-glomerular MTCs integrate broadly tuned glomerular inputs with archetypical, low amplitude reactivity

**Figure 6:**
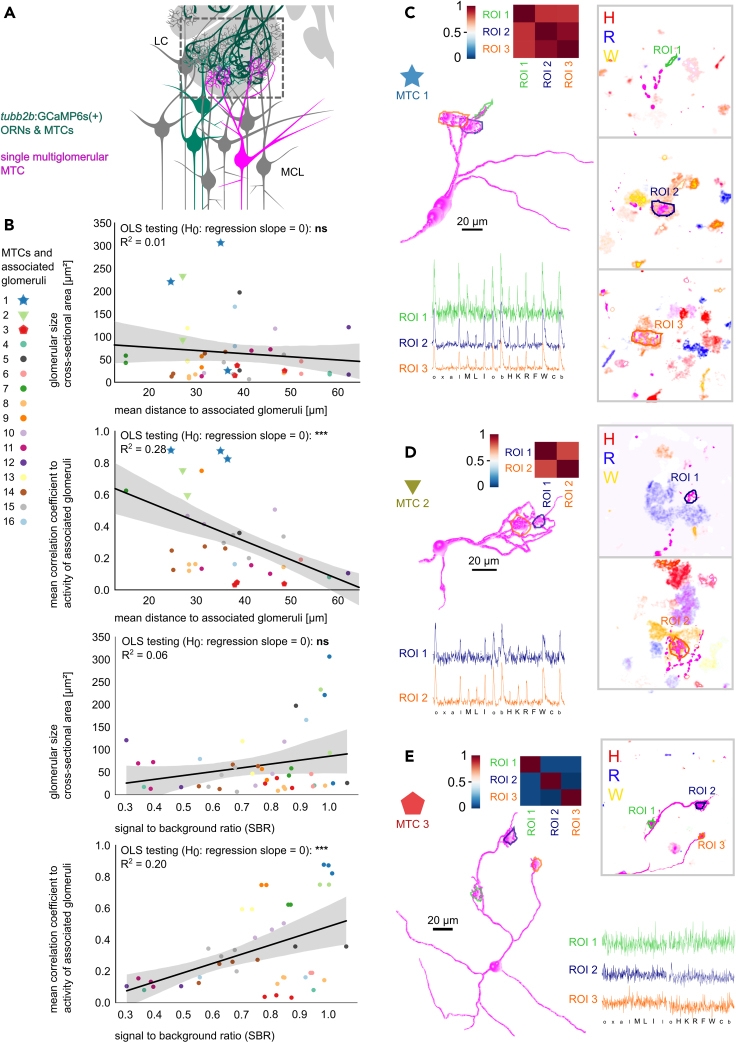
Positive correlation between activity patterns of glomeruli innervated by multi-glomerular MTCs decreases with inter tuft distance (A) Schematic of electroporation of a single, multi-glomerular MTC (magenta) in the lateral olfactory bulb of *tubb2b*:GCaMP6s tadpoles (dark green). (B) Scatterplots of properties of glomeruli associated with individual multi-glomerular MTCs (n = 16, color and symbols indicate MTCs). Shown are mean Euclidean distance of linked glomeruli plotted against the glomerular cross-sectional area and against the mean correlation coefficient between fluorescent time traces of connected glomeruli. Inverse relation between signal to background ratio and glomerular cross-sectional area or mean correlation coefficient between fluorescent time traces of connected glomeruli. Linear regression lines indicate negative or positive relations between the compared factors, gray shadings the confidence interval. ∗∗∗, p ≤ 0.001; ns, not significant, p > 0.05. (C–E) 3D Reconstructions representative for three types of multi-glomerular MTCs. Raw fluorescence traces of glomerular calcium responses (color indicates the region of interest), correlation matrices between the raw fluorescence traces (Pearson’s correlation coefficient) are shown. Inlays with individual image planes showing association of dendritic tufts (magenta) and reactive glomeruli (fluorescence intensity difference maps to amino acids, H, red; R, blue; W, yellow). Scale bar equals 20 µm. (C) A multi-glomerular MTC with highly correlated response profiles in glomerular regions. (D) MTC with similar odor tuning, but differences in lower amplitude responses. (E) MTC with no clear responses with amplitudes above noise level in the glomerular regions innervated by tufted dendrites (correlation coefficients around 0 between region time courses). Stimuli abbreviations see Figure 2A and methods section.

The high abundance of multi-glomerular MTCs in the lateral OB implied a potentially pivotal role in glomerular input integration. To investigate which odor information they integrate, we labeled individual multi-tufted MTCs (n = 16 MTCs) and measured amino acid evoked responses in associated glomerular input regions (n = 42; Figure 6). We found a weak, non-significant negative relation between (1) mean interglomerular distance and glomerular cross-sectional area and a significant, negative relation between (2) interglomerular distance and response profile similarity (Figure 6B). Especially the latter observation must be seen in context of the observed dependency between the signal-to-background ratio (SBR) of individual glomeruli and their (1) cross-sectional area and (2) mean correlation coefficient to associated glomerular response profiles (Figure 6B). We found three main functional types of multi-glomerular MTCs. First, MTCs with highly correlated response profiles and similar response amplitude differences (Figure 6C). Second, MTCs with similar dominant odor tuning, but differences in the lower amplitude responses (Figure 6D). Third, MTCs exhibiting low amplitude or no responses above noise level (Figure 6E).

**Figure 7:**
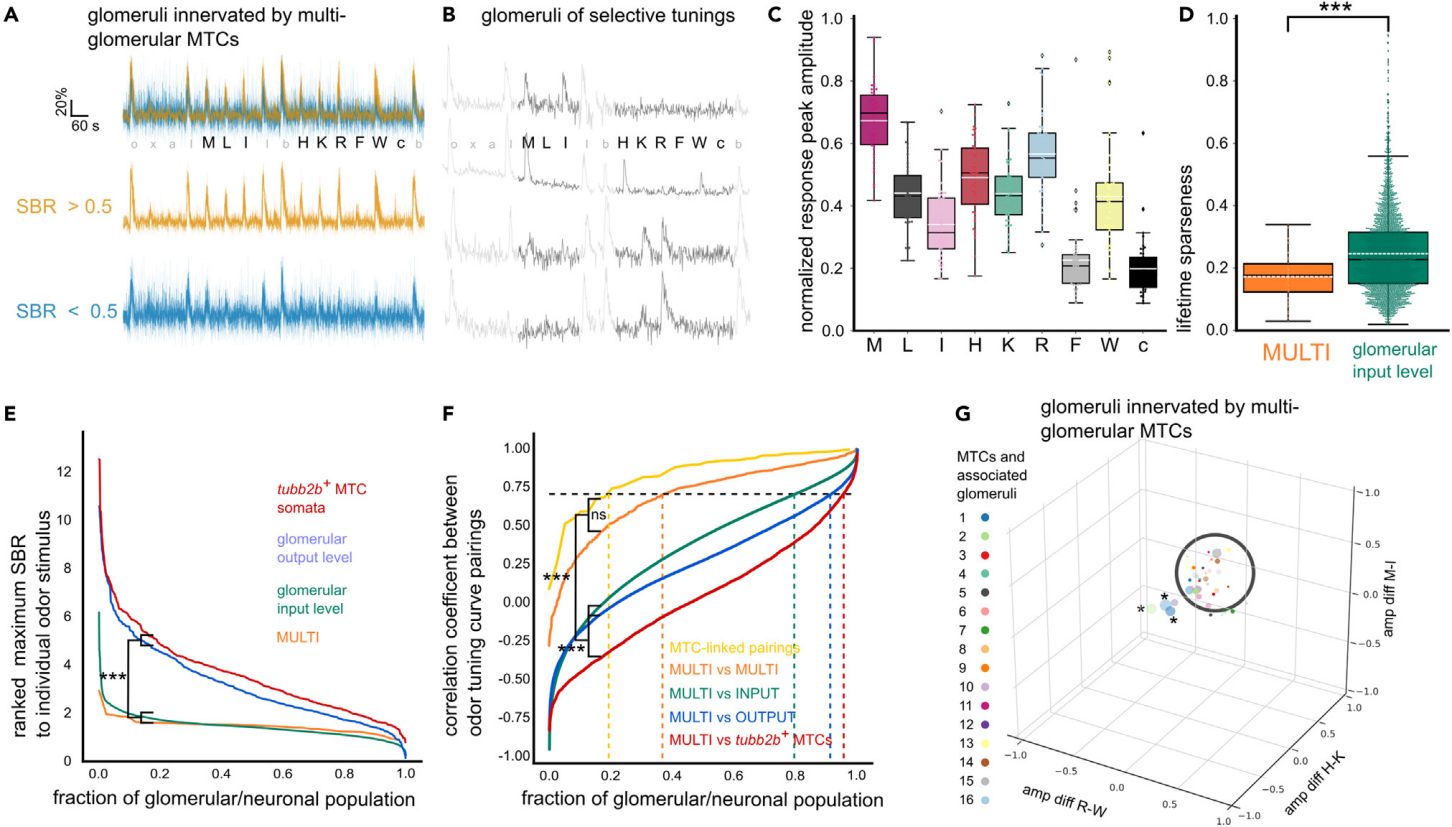
Glomerular inputs connected to multi-glomerular MTCs exhibit a broad underlying tuning profile (A) Overlay of odor responses of 42 glomeruli innervated by multi-glomerular MTCs (n = 16). Profiles are classified by their signal to background ratio (SBR; orange, SBR>0.5; blue, SBR<0.5). Response profiles show low amplitude responses to all amino acids applied except F. (B) Representative calcium time courses of glomerular input regions with selective tuning to individual amino acids. (C) Boxplots of amino acid response peak amplitudes of glomeruli innervated by multi-glomerular MTCs. Mean, dashed white line; median: black line; box outlines 1st and 3rd quartiles. (D) Boxplots of the lifetime sparseness of the MULTI dataset versus glomerular input level (green). Mean, dashed white line; median: black line; box outlines 1st and 3rd quartiles; ∗∗∗, p ≤ 0.001. (E) Ranked maximum SBR values to individual odor stimuli among multi-glomerular MTCs (MULTI, orange), glomerular input (green), output (blue), and MTC soma level (red). ∗∗∗, p ≤ 0.001. (F) Ranked distribution of correlation coefficients between all pairings of response peak amplitude vectors. Correlation coefficients were calculated for different datasets, i.e., glomeruli innervated by the same multi-glomerular MTC (MTC-linked), glomeruli of all multi-glomerular MTCs (MULTI), mixed glomerular input, output, and *tubb2b*^*+*^ MTCs. Dashed lines indicate Pearson’s correlation coefficient>0.7. ∗∗∗, p ≤ 0.001; ns, not significant, p > 0.05. (G) Glomeruli connected to multi-glomerular MTCs functionally cluster in a common region in odor space. Odor space is defined by relative amplitude differences between response to single amino acids. Note: Glomeruli of MTCs 1 and 2 (asterisks, see Figure 6) are outliers. See also Figure S7.

We pooled the fluorescent time courses of all glomeruli confined by dendrites of multi-glomerular MTCs for further analysis (Figure 7A). Independent of noise levels, all glomeruli were broadly responsive to amino acid stimuli. This was particularly evident when compared to the mixed population of previously recorded glomerular inputs with more selective tuning (Figure 7B, see also Figure 1). Glomeruli innervated by multi-glomerular MTCs had a broader tuning curve compared to glomeruli of the input level with unknown connectivity, but similar signal-to-background ratio (Figures 7C–7E). They are characterized by an underlying, broad, but characteristic amino acid response profile and consequently feature a highly significant lower lifetime sparseness (Figures 7C–7E).

As an indicator of tuning similarity within glomerular populations, we computed correlations of all possible pairings of response peak amplitude vectors. The ranked distribution of correlation coefficients between glomeruli innervated by the same multi-glomerular MTC (MTC-linked) was generally positive (Figure 7F). All glomeruli innervated by multi-glomerular MTCs (MULTI) featured also negative correlation coefficients, but generally had a strong bias toward positive values. Overall, tuning within this population was more similar than its similarity to the tuning of glomerular input, output, and *tubb2b*^+^ MTC populations. It is important to note that glomeruli of the MULTI group were compared to glomeruli that could be innervated either by uni- or multi-glomerular MTCs, since the respective wiring was unknown. The fraction of response peak amplitude vectors with high similarity to MULTI (>0.7) decreased from the input over the output to the MTC level (Figure 7F).

Glomeruli innervated by multi-glomerular MTCs also clustered tightly in our response amplitude-based analysis display of glomeruli and MTCs in odor space, where output level glomeruli and *tubb2b*^+^ MTCs occurred less frequently (Figure S7, see also Figures S2 and S3C). Outliers were glomeruli innervated by MTCs with a bias toward W on the R-W amplitude difference axis (Figure 7G, also shown in Figures 6C–6E).

### Individual MTCs can innervate glomeruli associated with bifurcating ORN axons

The morphology of ORN axonal arborizations^23^ and MTC tufted dendritic configurations seemed to follow similar patterns. Our functional recording of presynaptic glomerular responses associated with the tufted dendrites of multi-glomerular MTCs as well as functional recordings of postsynaptic glomerular responses associated with the axon terminals of bifurcating ORNs supported this idea: Diverging ORN axon terminals and converging MTC tufts can independently innervate glomerular neuropil with similar response profiles (Figures 4, 5, and 6).

**Figure 8:**
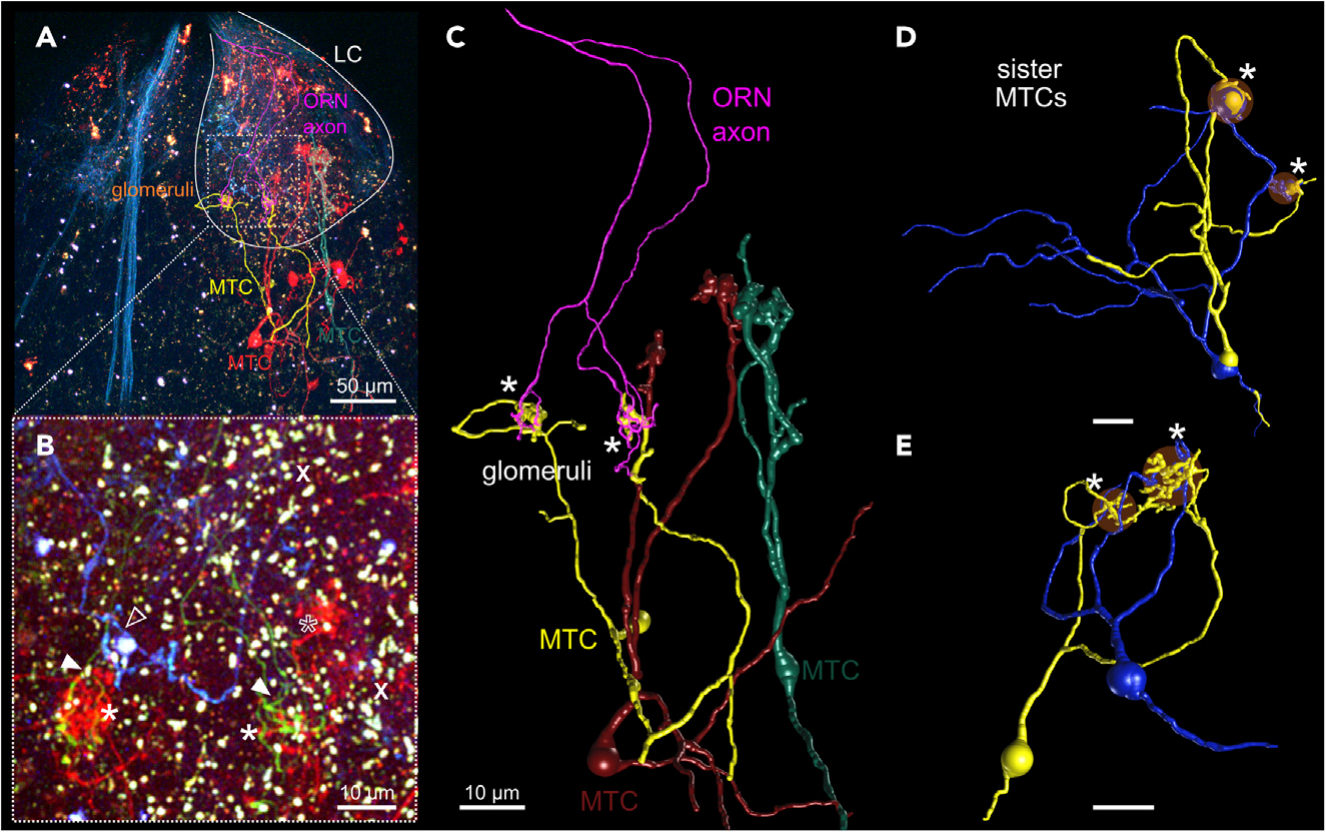
Multi-glomerular MTCs potentially re-converge input of anatomically distinct axon terminals of individual ORNs (A) Maximum intensity projection of the olfactory bulb with superimposed reconstructed ORN and MTC morphologies. In brainbow tadpoles, ORN axons that underwent CRE-mediated recombination are visible as green or cyan, fibrous structures. Scale bar equals 50 µm. (B) Close up of lateral glomerular cluster with colocalized axon terminals of a single ORN (green, arrowheads) and the two tufted dendrites of a single MTC (red, asterisks). Also visible are an additional, prominent ORN axon (cyan, empty arrowhead) and another tuft of a different MTC (empty asterisk). Note overrepresented autofluorescent, granular structures (white signal) due to contrast enhancement to visualize faint Cerulean and EYFP fluorescence. Scale bar equals 10 µm. (C) 3D reconstruction of an ORN (magenta) and an MTC (yellow) that innervate the same glomeruli with their two axon terminals/tufted dendrites and form a divergent-convergent glomerular module (pseudo-multiglomerular). Two additional labeled MTCs (red, green) are also shown. Scale bar equals 10 µm. (D and E) Reconstructions of multi-glomerular sister MTCs pairs (blue, yellow) that were labeled by sparse cell electroporation. They innervate the same set of glomeruli with their dendritic tufts (white asterisks). Note that glomeruli are sometimes very close and dendritic tufts are unequally arborized. Scale bar equals 10 µm.

We wanted to test the hypothesis, whether glomeruli innervated by bifurcating ORN axons could also be innervated by a specific set of MTCs. They could reconverge the information of both glomeruli with their glomerular dendrites or could integrate between different glomerular channels. To find connected pairs of diverging/converging neurons, we sparsely co-labeled ORNs and MTCs (Figure 8). We found sister MTCs that innervated the same pair of glomeruli with both their tufted dendrites (Figures 8D and 8E). Furthermore, we found one bifurcating ORN projecting into two distinct glomeruli that were convergently innervated by dendrites of a single MTC (pseudo-multi-glomerular wiring; Figures 8A–8C).

## Discussion

Basic elements required for olfactory glomerular map formation are orderly converging axons of receptor neurons expressing the same individual odorant receptor gene.^37^ The molecular receptive range of each odorant receptor is associated with a characteristic tuning to certain structural features of odor molecules.^32^^,^^38^ In the main OB, spatial grouping of glomeruli with similar receptive ranges forms the basis for a stereotypical and coarsely topographical functional odor representation.^12^^,^^13^^,^^31^

In the *Xenopus* lateral OB, the input or output level of glomeruli featured dominant responses to structural characteristics of mostly one or two individual amino acids, but numerous smaller responses were apparent. Even with a restricted set of odors, this led to a high diversity of response profiles in glomeruli as previously shown in ORN soma responses from OE slice preparations.^39^^,^^40^ Like zebrafish, glomerular responses in larval *X. laevis* have been shown to functionally cluster into distinct classes according to their tuning to amino acid structures.^14^^,^^26^^,^^33^^,^^41^ We observed a significant increase in tuning selectivity, lifetime sparseness and contrast for structural features from the glomerular input to the output level. This implied the existence of a feature-based sculpting of glomerular odor representations by the OB network.^26^^,^^29^^,^^42^

### No stereotypic or chemotopic arrangement among glomerular clusters

Non-delineated and often small glomeruli of similar tuning were distributed over the lateral OB and varied in number, identity and position between different animals, notwithstanding some topological bias. Non-selectively tuned glomerular species showed no clear topological arrangement at all. Overall, we did not find any hints for chemotopy or molecular feature clusters with gradual transitions or odor tuning similarity based on juxtaposition within the lateral cluster. However, chemotopy across the OB surface is also controversially discussed within the mammalian olfactory research and despite findings of glomerular arrangements based on functional groups or molecular features^12^^,^^13^^,^^31^^,^^36^^,^^43^ other studies have shown, that especially on the anatomically finer scale this arrangement can follow different principles or collapse entirely.^34^^,^^35^

This raises the question of how our findings compare to the OB organization of other aquatic vertebrates. The lateral olfactory subsystems of *Xenopus* and zebrafish have often been compared due to their shared cAMP-independent signal transduction cascade, amino acid sensitivity and association with V2R type odorant receptors and microvillous ORNs.^25^^,^^44^^,^^45^^,^^46^^,^^47^ In the lateral chain glomeruli of zebrafish, stereotypic, fractured spatial maps of amino acid-sensitive regions occur.^14^^,^^41^^,^^48^ Environmental enrichment with amino acids in young zebrafish alters the number and size of certain sets of glomeruli, whereas others are unaffected.^49^ This hints toward a certain flexibility in aquatic OB organization, at least in some glomerular modules. Our work focused mainly on the spatial component, but it is important to note that odor representations are highly transient and OB activity is shaped over time. On the MTC level, chemotopic odor representations are dynamically reorganized in the range of hundreds of milliseconds after stimulus application.^33^^,^^50^^,^^51^

### Coexistence of uni- and multi-glomerular modules as a source of odor map heterogeneity

The axonal and dendritic connectivity of a single glomerulus forms the basis of the functional OB organization and can be uni- or multi-glomerular.^18^^,^^20^ In zebrafish, ORNs and the majority of MTCs are only connected to a single glomerulus and thus OB organization is based on uni-glomerular modules.^18^^,^^52^^,^^53^^,^^54^^,^^55^ In *Xenopus*, ORN axons and MTCs regularly split and connect to more than a single glomerulus.^18^^,^^21^^,^^23^ In addition to these multi-glomerular modules, also uni-glomerular connectivity is found on both levels of the *Xenopus* OB.^23^^,^^24^ The organization of the olfactory network in amphibians of all developmental stages is primarily based on the coexistence of uni- and multi-glomerular connectivity with different hypothetical, overlapping wiring schemes.^18^^,^^19^^,^^23^^,^^56^

Uni-glomerular MTCs connecting to the same glomerulus exhibit highly correlated activity patterns,^24^^,^^57^ whereas multi-glomerular MTCs show lower correlation between their glomerular input activity patterns. Glomeruli selectively tuned to amino acids might be carried by uni-glomerular modules and arrange in a topological manner among the lateral cluster. The multi-glomerular modules possibly increase the variability in glomerular numbers and obscure the topology, due to the influence of a broad, archetypic amino acid response signature associated with glomeruli innervated by these MTCs. It is unclear how these information channels interact and multiple potential wiring strategies within and across the groups of uni- and multi-glomerular modules might exist.

We found that multi-tufted MTCs can convergingly connect to anatomically distinct glomeruli that are innervated by the bifurcating axonal branches of an individual receptor neuron. In this multi-glomerular wiring logic, incoming olfactory information is spatially split into two glomerular fractions and could still form an isolated transmission channel comparable to canonical uni-glomerular wiring strategies as in the rodent main OB.^4^^,^^58^ Nonetheless, this pseudo-multiglomerular wiring might potentially facilitate differential modulation of olfactory information in spatially segregated glomerular regions for downstream integration. This organization remotely resembles the rodent AOB with lack of stereotypy in glomerular number and position between animals.^7^^,^^8^ Multi-glomerular accessory mitral cells selectively integrate glomerular input in a heterotypic or homotypic manner resulting in a non-chemotopic but odorant receptor sequence and biological relevance-based odor map.^15^^,^^16^^,^^59^ We conclude that odor map heterogeneity in *Xenopus* arises from the variability of uni- and multi-glomerular innervation on both input and output level.

### Potential odorant receptor co-expression associated with multi-glomerular modules

An archetypical, low amplitude response profile in glomeruli innervated by multi-glomerular MTCs supports an underlying co-expression of a broadly amino acid–sensitive odorant receptor. Expression of more than one olfactory receptor could explain highly variable odor tuning classes observed in ORNs.^39^ Expression patterns of ancestral *v2r* genes and the broadly expressed *v2r-C* overlap in the lateral MOE of larval *Xenopus* and are associated with cAMP-independent, *trpc2*-positive, microvillous amino acid sensitive ORNs.^25^^,^^46^^,^^47^^,^^60^ In zebrafish, a similar lateral subsystem of microvillous ORNs expressing *trpc2* and V2R-type receptors is also amino acid sensitive.^14^^,^^44^ Amino acid responses are severely impaired in knockouts of a broadly expressed *OlfC* receptor, a *v2r-C* equivalent, supporting its role as co-receptor or chaperone.^61^ *OlfCc1* is required for detection of basic, aromatic, and neutral amino acids, while tuned to hydrophobic amino acids at a low sensitivity.^61^ In larval *Xenopus*, an ORN population could express a co-receptor leading to a baseline amino acid sensitivity that can be superimposed by the response profile of the main receptor. V2Rs are primary suspects for the glomerular cAMP-independent, amino acid sensitivity we observe. However, we cannot conclude a clear association of multi-glomerular innervation and V2R/co-receptor expression, since multi-glomerular wiring also exists in the cAMP-dependent, OR receptor expressing medial glomerular cluster.^25^ In fact, the general co-expression of multiple odorant receptors (OR, V1R, taars) might have led to the occurrence of multi-glomerular wiring strategies, possibly coinciding with the expansion of V1R/V2R and OR-type genes in amphibians.^19^^,^^37^ Multi-glomerular modules might be a prevalent phenomenon linked to ORN axon bifurcation in amphibians, but not necessarily exclusive to them.^17^^,^^18^ They could represent an intermediary type of neuronal module between fast, highly correlated uni-glomerular modules (latency coding of odor identity and concentration^24^^,^^57^) and slower, integrative multi-glomerular modules (detection of biologically relevant blends^15^^,^^62^). It is conceivable that a multitude of wiring logics emerges from the combination of singular and co-expression of odorant receptor types, increasing the coding capability of the olfactory system already at the OB level.

### Limitations of the study

Our stimulation approach aimed for a maximal activation of the glomerular array (Figure S8). Stimulation with high concentrations of odor molecules might obscure possible topologies of glomeruli very sensitive to individual odorants.^36^ Glomerular responses recorded in association with MTC tufted dendrites or ORN axon terminals represent examples of glomerular tuning and connectivity in the lateral cluster. Not only is the set of labeled cells random and limited in numbers, but also the finite set of stimuli limits the analysis of glomerular tuning. This limitation extends to the classification of homotypic or heterotypic innervation. It would be interesting to check if uni-glomerular modules show more spatial organization and chemotopy. Due to the lack of a specific marker, we were not able to only label uni-glomerular modules to differentiate population activity between uni- and multi-glomerular MTCs. Observations of the pseudo-multi-glomerular wiring pattern and sister MTCs innervating the same set of glomeruli were low in numbers due to the stochastic labeling approach (Figure 8). These observations should be seen as ‘proof of principle’ of an existing, unique wiring strategy and not as the generalized blueprint of glomerular modules. We investigated functional glomerular organization in larval animals, and it would be interesting to test how the OB is organized in post-metamorphic frogs.

## STAR★Methods

### Key resources table

**Table:** 

REAGENT or RESOURCE	SOURCE	IDENTIFIER
**Chemicals, peptides, and recombinant proteins**		

Wheat germ agglutinin (WGA), Alexa Fluor^TM^ 594 conjugate	Thermo Fisher Scientific	W1126
Oregon Green® 488 BAPTA-1 dextran, 10,000 MW	Thermo Fisher Scientific	O6798
Dextran, Cascade Blue^TM^, 10,000 MW	Thermo Fisher Scientific	D1976
Dextran, Alexa Fluor^TM^ 488, 10,000 MW	Thermo Fisher Scientific	D22910
Dextran, Alexa Fluor^TM^ 568, 10,000 MW	Thermo Fisher Scientific	D22912
Dextran, Alexa Fluor^TM^ 594, 10,000 MW	Thermo Fisher Scientific	D22913
CellTrace™ Calcein Violet, AM	Thermo Fisher Scientific	C34858
CellTrace™ Calcein Green, AM	Thermo Fisher Scientific	C34852
CellTrace™ Calcein Red-Orange, AM	Thermo Fisher Scientific	C34851
Fluo-4, AM, cell permeant	Thermo Fisher Scientific	F14201
Dimethylsulfoxid (DMSO)	Merck (before Sigma-Aldrich)	Cas#67-68-5;
Pluoronic F-127 (10% in water)	Biotium	Cas#9003-11-6
MK-571, sodium salt	Enzo (before Alexis Biochemicals)	Cas#115104-28-4
Tricaine (MS-222)	TCI chemicals	Cas#886-86-2
L-Amino acids (analytical standard) L-Alanine, L-Arginine Hydrochloride, L-Cysteine hydrochloride, Glycine, L-Histidine hydrochloride, L-Isoleucine, L-Leucine, L-Lysine hydrochloride, L-Methionine, L-Phenylalanine, L-Serine, L-Threonine, L-Tryptophan, L-Valine	Merck (before Sigma-Aldrich)	LAA21 Cas#56-41-7, Cas#1119-34-2, Cas#52-89-1, Cas#56-40-6, Cas#5934-29-2, Cas#73-32-5, Cas#61-90-5, Cas#657-27-2, Cas#63-68-3, Cas#63-91-2, Cas#56-45-1, Cas#72-19-5, Cas#73-22-3, Cas#72-18-4
Bile acids Taurocholic acid, Glycocholic acid, Cholic acid, Deoxycholic acid	Merck (before Sigma-Aldrich)	Cas#345909-26-4, Cas#1192657-83-2, Cas#81-25-4, Cas#83-44-3
Alcohols, Aldehydes, Ketones α-Terpineol, β-ionone, β-phenylethyl alcohol, γ-phenylpropyl alcohol, Citral	Merck (before Sigma-Aldrich)	Cas#98-55-5, Cas#14901-07-6, Cas#60-12-8, Cas#122-97-4, Cas#5392-40-5
CleanCap® Cre mRNA (5moU)	TriLink Biotechnologies	Cas#L-7211

**Experimental models: Organisms/strains**		

Xla.Tg(tubb2b:Katushka; cryga:Venus)	The European *Xenopus* Resource Center	RRID:EXRC_0202
Xla.Tg(tubb2b:GCaMP6s; Rno.elas:GFP)	The National *Xenopus* Resource	RRID:NXR_0107
Xla.Tg(CMV:Brainbow-1.0H)	Livet et al.,^63^	RRID:NXR_0001

**Software and algorithms**		

CaImAn	Giovannuci et al., 2019	RRID:SCR_021152
Fiji	Schindelin et al.^64^	RRID:SCR_002285
Vaa3D	Peng et al., 2010	RRID:SCR_002609
Inkscape	Inkscape project	RRID:SCR_014479

**Other**		

Cyclo II peristaltic pump	Roth	EP76.1
MilliManifold^TM^ 16 to 1 channel	ALA Scientific Innstruments	MilliManifold^TM^ 16
8-channel, gravity perfusion system with pinch valve controllers	ALA Scientific Innstruments	VC^3^-8PG
Borosilicate microcapillaries	Warner Instruments	2BF150-86-10
Micropipette Puller P-1000	Sutter Instrument	RRID:SCR_021042
Micropipette Beveler	World Precision Instruments	Micropipette Beveler 48000
Femto Jet express microinjector	Eppendorf AG	Femto Jet express
Electroporator	NPI Electronics	ELP-01D
Axoporator® 800A	Axon Instruments/Molecular Devices	Axoporator 800A
Micromanipulator for injection setup	Scientifica	PatchStar
Fluorescence stereomicroscopes	Olympus	BX51WI; SZX16
Fluorescent light source	Andor Technology-Oxford Instruments	AMH-200-F6S
Fluorescent light source	Excelitas technologies (before: Lumen Dynamics)	X-Cite 120Q
Multiphoton microscope	Nikon	A1R MP; RRID:SCR_020319

### Resource availability

#### Lead contact

Further information and requests for resources and reagents should be directed to and will be fulfilled by the lead contact, Thomas Hassenklöver (thomas.hassenkloever@physzool.bio.uni-giessen.de).

#### Materials availability

This study did not create any new unique reagents.

### Experimental model and study participant details

#### Ethical statement

All experiments followed the guidelines of laboratory animal research and were approved by the regional board (RP Giessen; Az: V54-19c2015h01 GI 15/7) and the Niedersächsisches Landesamt für Verbraucherschutz und Lebensmittelsicherheit, Oldenburg, Germany (Az: 16/2136) following the German animal welfare law and the European legislation for the protection of animals used for scientific purposes (2010/63/EU).

#### Background and developmental stages

In this work we used larval *Xenopus laevis* albino (wild-type; NASCO strain) or transgenic tadpoles all of developmental stages 50–53.^65^ Health status and lack of developmental deformations was assessed beforehand. The sex of the animals was not determined.

#### Transgenic lines used

• Xla.Tg(*tubb2b*:Katushka; cryga:Venus), RRID:EXRC_0202
• Xla.Tg(*tubb2b*:GCaMP6s; Rno.elas:GFP), RRID:NXR_0107
• Xla.Tg(*CMV*:Brainbow-1.0H), RRID:NXR_0001

#### Availability

All lines were received from the European *Xenopus* Resource Center Portsmouth (EXRC, UK) and the National *Xenopus* Resource (NXR, Woods Hole, USA). The lines used in this study are also kept in the animal facility of the Institute of Animal Physiology in Gieβen and oocytes/embryos can be provided upon reasonable request.

#### Housing and husbandry

Animals were fed daily with *Spirulina* and *Chlorella* (MS-Tierbedarf, Gieβen Germany, Dohse Aquaristik GmbH, Bonn, Germany) reared as schools in aquaria of 2–7.5 L at a water temperature of 22°C under 12 h dark/light cycles in our breeding facility in Göttingen/Gieβen.

### Method details

#### Anesthesia and whole-mount preparation

We anesthetized larval *Xenopus laevis* in 0.02% tricaine/MS-222 solution (pH 7.6 in tap water) until complete irresponsiveness.^66^ We killed anesthetized animals by severing the nervous system at the brainstem. A rectangular tissue block from the upper jaw, including the peripheral olfactory system plus olfactory bulbs, was excised for whole-mount preparations. Connective tissue occluding the nostrils and superincumbent palatial tissue on the ventral olfactory bulb was removed using forceps and fine scissors. For imaging experiments, the whole mount preparation was placed in a recessed recording chamber and stabilized by a nylon-stringed platinum grid.^66^^,^^67^

#### Wheat germ agglutinin dye-based glomerular labeling

We removed residual water in the nostrils of anesthetized tadpoles with microfiber tissue and gradually applied ∼1 μL of 250 ng/μL WGA-Alexa Fluor 594 (Thermo Fisher, dissolved in frog ringer) using micro loader pipette tips (Eppendorf, Microloader 25 μL). Gently pipetting the dye solution up and down assured homogeneous distribution in the nasal cavities (Figure S1E). After being kept in a moistened chamber for 10 min, we washed off excess dye solution and put the anesthetized tadpoles back to their aquaria to recover from anesthesia.

#### Dextran dye based labeling of glomerular cluster

Prior to sparse-cell electroporations of MTCs, we labeled ORNs via bulk electroporation of fluorophore-coupled dextrans.^68^ After anesthesia, dextran-dye crystals (Oregon green BAPTA dextran, MW 10,000; 3 mM in frog saline) were dissolved in residual water in the nostrils. We positioned 0.2 mm platinum wire electrodes and applied four/six (stage 50–51/52–53) sets of three square-pulses, alternating the polarity after each set (15/20 V, 500 ms duration and 25 ms delay; NPI Electronics; 3 μF capacitor connected in parallel). We performed the procedure on both nostrils with one electrode touching the animal laterally of the ON. Anesthetized animals were transferred to aquaria for recovery and used for experiments after 1–3 days.

#### Sparse cell labeling of ORNs using dextran dyes

We labeled sparse sets of ORNs via electroporation^69^ (Figure S1F). Micropipettes with small tapers (8–15 MΩ) were pulled (P-1000, Sutter Instruments) from borosilicate glass capillaries (outer diameter 1.5 mm, inner diameter 0.86 mm, length 100 mm, with filament; Warner Instruments). Micropipettes were filled with 3–4 μL of fluorophore-coupled dextran solution (Alexa Fluor 594/568 dextran; 10 kD, 3 mM in frog ringer, Thermo Fisher) and mounted to the head stage of a single cell electroporator (Axoporator 800A, Axon Instruments/Molecular Devices). We positioned the anesthetized animal in a Petri dish, covered with a moistened microfiber tissue and in contact with the reference electrode. Under a microscope (Olympus SZX16; light source: X-Cite Series 120 Q, Excellitas technologies), the micropipette was penetrated into the lateral part of the main olfactory epithelium using a micromanipulator (Scientifica, PatchStar). A train of voltage pulses was applied (50 V, 300 μs pulse length, 500 ms train duration, 300 Hz). Fluorescent cell bodies were visible a few seconds after successful electroporation. Animals were transferred to aquaria for recovery from anesthesia and used for experiments after one to three days.

#### Sparse cell labeling of MTCs using dextran dyes

Whole-mount preparations were placed under a microscope with fluorescent illumination (Olympus BX51WI; light source AMH-200-F6S; Andor Technology-Oxford Instruments). Micropipettes filled with dextran-coupled fluorophores (see above; 3 mM in frog ringer, Alexa Fluor 488/555/594 and cascade blue dextran, 10 kD, Thermo Scientific) were mounted to the head stage of a single cell electroporator. We lowered the pipette tip toward the OB surface using a micromanipulator before penetrating it into the mitral cell layer and applying a voltage pulse train (50 V, 300 μs pulse length, 500 ms train duration, 300 Hz; details see.^68^ We used prior bulk electroporation with fluorophore-coupled dextrans, WGA application, or the Katushka fluorescence of the *tubb2b* transgenic reporter line^30^ to identify the mitral cell layer.

#### Sparse ORN labeling in transgenic brainbow tadpoles

Fluorescent protein expression was induced in multiple ORNs by injection of CRE recombinase mRNA into the MOE of transgenic *CMV*:Brainbow tadpoles with subsequent bulk electroporation (Figures S1H–S1I). We fabricated microinjection pipettes for CRE mRNA injection from borosilicate glass capillaries (outer diameter 1.0 mm, inner diameter 0.58 mm, length 100 mm; Warner instruments) that we then sharpened at an angle of 20–30° (Micropipette Beveler 48000; World Precision Instruments). CleanCap NLS-Cre Recombinase mRNA solution (100 ng/μL; TriLink Biotechnologies) was mixed 10:1 with cascade blue or Alexa Fluor 594 dextran (3 mM in frog ringer, 10 kDa, Thermo Fisher) and stored on ice. We mounted micropipettes filled with mRNA solution to a micromanipulator connected to a pressurized microinjector (FemtoJet, Eppendorf). Brainbow tadpoles were anesthetized and transferred to a moistened silicone rubber-filled Petri dish. We penetrated the pipette into the mucosa and increased the supply pressure until dye extrusion was visible. The pressure was increased by small increments (100–1000 hPa) until the dye slowly spread in the surrounding tissue. This was repeated at up to five injection sites in the main olfactory epithelium. Immediately after injection, we performed bulk electroporation (as described above). Tadpoles were transferred back to aquaria and left for recovery. Recombination-mediated cyan/yellow fluorescent protein expression was detectable in ORNs after a few days, axonal staining in the OB after three to five weeks.

#### Loading of MTCs with AM-dyes/calcium indicators

For functional experiments, we injected calcium indicator solution into the ventral OB (Figure S1G, Offner et al., 2020). Fluo-4 AM (50 μg, Thermo Fisher) was dissolved in DMSO (Sigma-Aldrich) and mixed with 10 μL Pluronic F-127 (Biotium) and 35 μL frog ringer. After centrifugation at 16100*g* for 60 s, we mixed the supernatant with 0.3 μL cascade-blue dextran (3 mM in frog ringer; 10 kDa) and 3 μL MK571 (50 μM in frog ringer; Alexis Biochemicals). Micropipettes (see above) were filled with 3 μL calcium indicator solution and mounted to a head stage with a manual pressure supply system (syringe with three-way valve). The micropipette was pierced into the lateral mitral cell layer using a micromanipulator under a microscope with fluorescent illumination (Olympus BX51WI; light source AMH-200-F6S; Andor Technology-Oxford Instruments). The pressure was built up in the syringe by manual compression and released by opening the valve. Dye extrusion into the OB was observed, and the applied pressure was adjusted if necessary to gradually load the tissue surrounding the injection site for a maximum of 5 s. We performed three injections at different sites in the lateral mitral cell layer to achieve visible Fluo-4 fluorescence in neurons of interest.

For multicolor morphological labeling of OB neurons and their dendritic projections, three differently colored Calcein AM dye solutions (Calcein Green AM, Calcein Orange AM, Calcein Violet AM; Thermo Fisher; prepared like calcium indicator mix without Cascade blue-dextran) were injected at three sites of the lateral mitral cell layer to label partially overlapping OB territories (Figures 6A, 6E, S1D, and S1E).

#### Multiphoton microscopy and imaging settings

We obtained 3D-image stacks of the OB with a multiphoton microscope (Nikon A1R MP). The excitation wavelength was 800 nm for Fluo-4/Alexa Fluor 568/594, 780 nm for Alexa Fluor 488/555/594, cascade blue, Oregon Green BAPTA, Calcein Green/Orange/Violet, Katushka, 900 nm for GCaMP6s, 850 nm for Cerulean and EYFP. Fast volumetric calcium imaging experiments were performed using the resonant scanner unit (25–35 image planes, 512 × 512 px), x, y resolution glomerular recordings: 0.25 μm/px: x, y resolution mitral cell layer recordings: 0.5 μm/px; z-interplane distance of 4–6 μm at 0.5–1 Hz.

#### Odor stimuli and odorant application system

In all calcium imaging experiments, individual basic (H, K, R), aromatic (F, W), and long-chain neutral (M, L, I) L-amino-acids and mixtures of those were used (b: basic aromatic mix: H, K, R, F, W; long-chain neutral mix l: M, L, I, V; short-chain-neutral mix s: A, C, S, T). All amino acids/mixtures were prepared from 10 mM frozen stocks and applied at final concentrations of 100 μM in frog ringer. In addition, we used a mixture of alcohols and aldehydes (α-Terpineol, β-ionone, β-phenyl-ethyl alcohol, γ-phenyl propyl alcohol, citral; 50 μM in frog ringer) known not to activate the lateral OB and frog ringer as control stimuli. In the GCaMP6s recordings, we used a bile acid (x) and amine mixture (a) as additional negative controls^25^ and a mixture of all odorants (o, 25 mM).

We applied odorants to the olfactory epithelia of the whole-mount preparations for 5 s via two gravity-fed eight-channel perfusion systems (ALA-VM-8 Series; VC3-8xP Series; ALA Scientific) connected via silicone tubing to a shared outflow (Milli Manifold; 16 inlet ports; ALA Scientific) positioned in front of the nostril. Without stimulations, constant perfusion of frog ringer (98 mM NaCl, 2 mM KCl, 1 mM CaCl_2_, 2 mM MgCl, 5 mM Na-pyruvate, 5 mM glucose, 10 mM HEPES, pH 7.8, osmolarity of 230 mOsmol/l) was present, and excess solution drained by a peristaltic pump (Cyclo II, Roth). We recorded for 15 s before each stimulus application. We chose inter-stimulus intervals of 1 min to avoid desensitization effects. Each experiment consisted of at least two pairs of stimulus sequences to eight stimuli. There was one repetition of the respective amino acid/odorant mix in each sequence at the beginning and end of the recording. We used all four sequences in MTC somatic recordings and only one pair of sequences in glomerular recordings for CaImAn analysis. Detailed schematics of the different experimental protocols are depicted in Figures S1A–S1C.

### Quantification and statistical analysis

#### Imaging data pre-processing

We corrected 5D calcium imaging data (two color channels, x, y, z, and time dimension) for a line shift originating from bilinear scanning. During the 16/32 min recordings under perfusion, x, y, and z drift occurred in all measurements. We merged multiple subsequent z-planes (4/6 planes, interplane distances 4–6 μm) to one virtual plane (20–30 μm) by maximum intensity projection to compensate z drift. While losing z-resolution with this method, spatial footprints were not easily lost due to z-or x-y drift later. We used a piecewise-rigid motion correction algorithm (NoRMCorre) to obtain lateral drift-corrected image stacks and subsequent plane-wise denoising, deconvolution, and de-mixing of the data by CaImAn.^70^^,^^71^^,^^72^^,^^73^

#### ROI extraction and neuronal activity visualization

We used CaImAn’s constrained non-negative matrix factorization (CNMF) to extract spatial footprints and their temporal components.^72^ To visualize stimulus-induced neuronal activity, we created fluorescence intensity difference maps. Therefore, we subtracted the mean of the maximum fluorescence values of the calcium transients and the two neighboring frames (8 s after stimulation onset) from the mean baseline fluorescence (5 time frames: 5 s prior to stimulus; see also Figure S1A). We used the deconvolved and gaussian-filtered data for intensity difference maps to visualize only ROIs detected by CaImAn. In this approach aiming to display glomerular/neuronal tuning in a qualitative manner, we used the resulting individual (response peak) intensity difference image (grayscale) for each individual stimulus and merged those images in Fiji with individual stimulus specific primary colors as multicolor stacks, mostly the maximum intensity z-projection. If not stated otherwise, images display the olfactory bulb in a rostro-caudal (top to bottom) and latero-medial (left to right) orientation. Spatial footprints of ROIs were extracted by CaImAn together with their size, positional information in the imaged 3D volume, and corresponding fluorescent time courses (raw, denoised, and deconvolved).

#### Filtering of ROIs and their fluorescent signals

ROIs were automatically excluded if

1. The spatial footprint was below 25 μm^2^ (based on minimum glomerular diameters observed: 5–40 μm)^23^^,^^26^^,^^28^
2. Stimulus-evoked fluorescence intensity peaks did not exceed 3x the standard deviation of the non-stimulus response intervals (25 s after stimulation for 15 s), and
3. Not at least two responses (i.e., mix and single amino acid) were present.

We manually excluded ROIs if

1. Their deconvolved and raw fluorescent time traces did not match (peaks missing),
2. Their position was out of selected boundaries (e.g., not part of the glomerular cluster or mitral cell layer), and
3. Their fluorescence increase was of non-neuronal origin, e.g., ROI position was not inside the glomerular layers.

All remaining raw fluorescence time traces were baseline corrected by asymmetric least squares smoothing,^74^ absolute fluorescence changes transformed into ΔF/F,^26^ and the time trace of each ROI normalized to the maximum fluorescent (response) amplitude found in the time series (range of normalized fluorescence values: 0–1).

#### Essential parameters for odor tuning analysis

We analyzed the calcium responses of glomeruli and neurons on the glomerular input, output, and MTC soma level according to their tuning to the eight single amino acid stimuli (M, L, I, H, K, R, F, W). We used the stimulus-response peak amplitude of the normalized ΔF/F fluorescent time traces (in the interval 15 s after stimulus application start) as a measure of odor-evoked response intensity. For all stimuli that were applied multiple times, the maximum response amplitudes were averaged.

#### Odor tuning

We analyzed all glomeruli and neurons according to a ‘default’ and a ‘dominant odor tuning’. We defined the default/dominant odor tuning as the set of stimuli triggering calcium responses (peak amplitude) above 2x/3x the standard deviation of averaged baseline fluorescence of the entire stimulation sequence (15 s intervals, 25 s after stimulus onset). For assignment of these tunings to each glomerulus/neuron, we normalized each fluorescent time trace by its maximum fluorescence intensity value (as described above). The default/dominant odor tuning of glomeruli/neurons is presented as a sequence of the single-letter amino acid codes. A region with dominant odor tuning HFW exhibits suprathreshold calcium responses to H, F, and W, and all potential peaks below 3x SD of the baseline fluorescence are neglected (Figure 2A). We linked 21 of the most frequent default/dominant odor tunings with particular colors (Figures 2E, 2F, and S4A).

#### Correlation and response amplitude (difference) based analysis

We calculated the pairwise differences (ranging from −1 to 1) between the normalized response peak amplitudes to amino acid stimuli. Among others, those amplitude differences were used as x, y, and z coordinates of individual ROIs in a simplified 3D odor space representation (for details see Figure S2).

We normalized the response peak amplitudes for odor tuning analysis to the maximum response evoked by the selected eight stimuli (M, L, I, H, K, R, F, W; range 0–1). Those amplitude response vectors were used for correlation matrices (Pearson’s correlation coefficient), hierarchical cluster analysis (average linkage method, using correlation distances), and (lifetime) sparseness calculation.^75^ Lifetime sparseness equals 0, when a region responds equally strongly to all stimuli and 1, when exclusively responding to one. N represents the length, and r_n_ the nth response of the amplitude response vector.

$$sparseness=\frac{1-{\left(\frac{\sum r_{n}}{N}\right)}^{2}∕\left(\sum \frac{r_{n}^{2}}{N}\right)}{1-\frac{1}{N}}$$

We calculated the normalized distances between the columns of the hierarchical cluster diagram (cluster distances; 0–1) as a measure of how individual stimuli were linked on the level of their amplitude response vectors.

#### Signal to background/noise ratio

We calculated the signal to background ratio (SBR), where (ΔF/F)_s_ is the mean fluorescence signal of 15 s after stimulation, (ΔF/F)_ns_ the mean fluorescent signal of the 15 s baseline intervals, 25 s after stimulus onset, and σ_s_^2^/σ_ns_^2^ the respective variances.

$$SBR=\frac{{\left(\Delta F∕F\right)}_{s}-{\left(\Delta F∕F\right)}_{ns}}{\sqrt{0.5\times \left(\sigma_{s}^{2}+\sigma_{ns}^{2}\right)}}$$

#### Normalized spatial territories of glomerular species

We normalized glomerular/neuronal positions according to their relative positioning along the medio-lateral, caudo-rostral, and dorsoventral axis. Therefore x, y, and z coordinates were normalized to manually defined coordinates at the respective boundaries of the glomerular cluster/mitral cell layer. For the glomerular output layer, we used WGA-Alexa Fluor 594 to label all glomeruli *in vivo* and high z-resolutions during image acquisition to ensure full coverage of the lateral cluster and its glomeruli in all animals. On the glomerular input layer, faint green GCaMP6s expression marked the outlines of the lateral cluster. Centroids of the positioning of individual glomerular species (glomeruli of a specific default/dominant odor tuning) were calculated from the mean x, y, and z coordinates along each of these normalized axes. Ellipsoids of 1x SD along each axis with the centroid coordinate as origin served to estimate the territories of each glomerular species.

#### Measures of juxtaposition and chemotopy

We calculated the Euclidean distances between the positional centroids of different glomerular species as a measure of juxtaposition. For chemotopy analysis, centroids of glomerular species dominantly tuned to two or three amino acid stimuli were used (compound profiles, e.g., KR or HFW). We calculated their positional deviation from the centroid of the two or three glomerular species dominantly tuned to the individual stimuli of the compound profile only (e.g., K and R or H, F and W) by Euclidean distance. The smaller this distance, the more centered were the glomerular species with a compound profile between the glomerular species tuned to the individual stimuli only.

We defined glomeruli tuned to (combinations of) H, F, and W; M, L and I, or K and R as glomeruli tuned to structurally similar amino acids (similar molecular receptive range).

#### *In silico* structural similarity scoring

We used the online tool CheMineTools to calculate Tanimoto scores of maximum common substructure (MCS) and atom pair distance (AP) as a quantitative descriptor of structural similarity between amino acid pairings.^76^ Tanimoto scores were calculated using the reference numbers of the amino acids: H: 6274, W: 6305, F: 6140, R: 6322, K: 5962, M: 6137, I: 6106, and L: 6306.

#### MTC morphological analysis

MTCs labeled via sparse/single cell electroporation in the MOB of larval *Xenopus laevis* were semi-automatically reconstructed from two-photon image stacks using Vaa3D.^56^^,^^77^ The reconstructions were translated into a hierarchical tree-structure by defining branching and endpoints. The cell soma was defined as root segment of the tree, with each segment connecting to exactly one parent segment. The DBSCAN algorithm (Density-Based Spatial Clustering of Applications with Noise) implemented in the Python scikit-learn machine learning package was used to determine the number of tuft-clusters in the tree structure.^78^ These were defined as clusters of at least five end- and/or branching points within a radius of 15 μm. All endpoints outside these clusters were counted as blunt neurite endings. Distances between soma and tufts were measured along the dendritic branches and distances between tuft-clusters were measured as Euclidean distance of their center points. Tuft volume was calculated as convex hull around all end- and branching points of individual tuft-clusters. Nine morphological parameters were used for a principal component analysis. The values of each parameter were standardized by subtracting the mean and scaling to variance.

#### MTC primary dendritic projection analysis

We injected three differently colored Calcein AM dyes into the MCL of the lateral olfactory bulb (details see prior section and Figure S1D). Dye taken up by the MTC somata (MCL) was visible also in the MTC primary tufts in the glomerular layer (GL). To evaluate the positions of primary dendritic tufts of defined, anatomically distinct MTC subpopulations, we used binary thresholding of voxels in multi-color 3D image stacks of Calcein dye injections. Voxels with a color ratio of a defined primary color (red, green or blue; from Calcein Orange, Green and Violet injection) was at least 2:1 to both other primary colors were used to classify MTC somata and primary tufts according to color categories (MCL-red, green and blue and GL-red, green and blue). Spatial distributions of pixels of those three categories along the x, y and z axis were compared by manually selecting either the pixels of the GL only (clearing pixels beyond the GL manually in Fiji) or all remaining pixels of the OB sample except the ones in the GL. Spatial distributions of pixels of the respective color category of the MCL and GL were compared along the x- (medio-lateral), y- (rostro-caudal) and z (dorsoventral) axes using boxplots or 2D kernel-density-estimate plots (excluding the z axis).

#### Statistical analysis

Statistical analysis was performed with the Python scipy.stats module.^79^ The Shapiro-Wilkins test was used to assess whether individual datasets had an underlying normal distribution. If not stated otherwise, we used Kruskal-Wallis two-sided testing for comparisons between more than two groups and subsequent post-hoc Dunn tests with Bonferroni correction for pairwise comparisons. For comparison between two groups, we used Mann-Whitney-U testing. Linear regression was assessed using ordinary least squared (OLS) testing of H_0_: slope of linear regression line computed for data equals 0. No prior sample size estimation or randomization approach was performed. n represents the number of animals (i.e., olfactory system explants) used, if not indicated otherwise.

## Data Availability

• Raw, processed, and intermediate multiphoton imaging data and morphological reconstructions reported in this paper will be shared by the lead contact upon request. • All code is available as of the date of publication upon request. • Any additional information required to reanalyze the data reported in this paper is available from the lead contact upon request.
