# Supplementary material for: Functional odor map heterogeneity is based on multifaceted glomerular connectivity in larval Xenopus olfactory bulb
Source: iScience. 2023 Aug 2;26(9):107518. doi: 10.1016/j.isci.2023.107518 (PMC10448113; doi:10.1016/j.isci.2023.107518)
Supplement: Document S1. Figures S1–S8 [file mmc1.pdf]

iScience, Volume *26*

Supplemental information

Functional odor map heterogeneity is based on multifaceted glomerular connectivity in larval *Xenopus* olfactory bulb

Thomas Offner, Lukas Weiss, Daniela Daume, Anna Berk, Tim Justin Inderthal, Ivan Manzini, and Thomas Hassenklöver

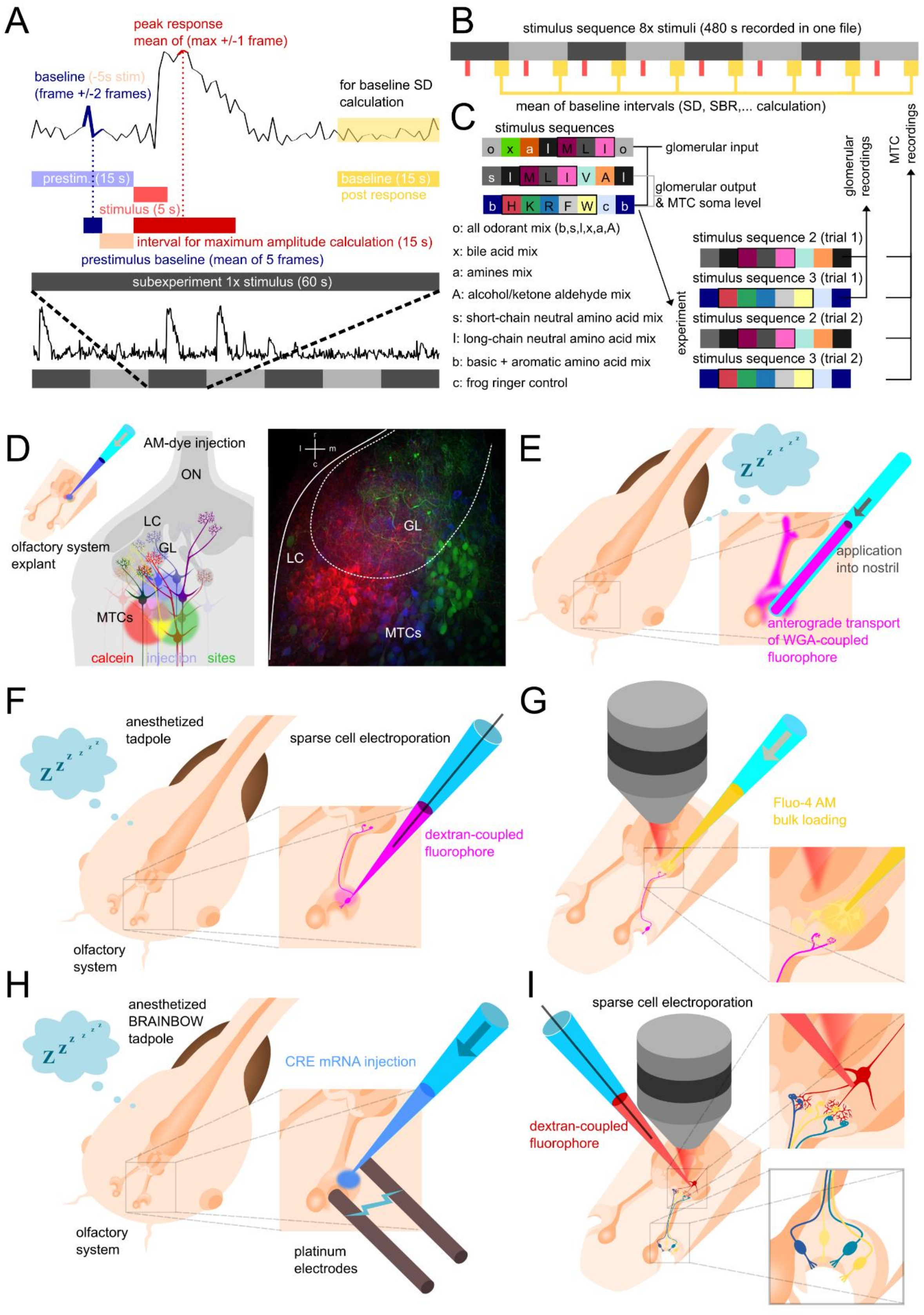
A
peak response
mean of (max +/-1 frame)
baseline (-5s stim)
(frame +/-2 frames)
for baseline SD
calculation
prestim. (15 s)
stimulus (5 s)
baseline (15 s)
post response
interval for maximum amplitude calculation (15 s)
prestimulus baseline (mean of 5 frames)
subexperiment 1x stimulus (60 s)
B
stimulus sequence 8x stimuli (480 s recorded in one file)
mean of baseline intervals (SD, SBR,... calculation)
C
stimulus sequences
glomerular input
glomerular output
& MTC soma level
glomerular
recordings
MTC
recordings
o: all odorant mix (b,s,l,x,a,A)
x: bile acid mix
a: amines mix
A: alcohol/ketone aldehyde mix
s: short-chain neutral amino acid mix
l: long-chain neutral amino acid mix
b: basic + aromatic amino acid mix
c: frog ringer control
experiment
stimulus sequence 2 (trial 1)
stimulus sequence 3 (trial 1)
stimulus sequence 2 (trial 2)
stimulus sequence 3 (trial 2)
D
AM-dye injection
ON
LC
GL
olfactory system
explant
MTCs
calcein
injection
sites
LC
GL
MTCs
E
application
into nostril
anterograde transport
of WGA-coupled
fluorophore
F
anesthetized
tadpole
sparse cell electroporation
dextran-coupled
fluorophore
olfactory
system
G
Fluo-4 AM
bulk loading
H
anesthetized
BRAINBOW
tadpole
CRE mRNA injection
olfactory
system
platinum
electrodes
I
sparse cell electroporation
dextran-coupled
fluorophore

Figure S1. Schematics of experimental approaches, Related to Figure 1

(A) Parameters and intervals of the stimulation protocol/sequence. Odor stimulation of 5 s (light red) after a 15 s prestimulus interval (light blue). For intensity difference maps, the mean of five prestimulus baseline frames (dark blue; 5 s prior to stimulus onset) are subtracted from the mean of the peak response amplitude +/- 1 neighboring frame. For all other amplitude-based methods, the response peak amplitude is defined as the peak response found in the 15 s interval after stimulus onset (dark red). Baseline intervals for standard deviation or signal-to-background ratio are calculated from 15 s post response intervals (25 s after stimulus onset; calculated across multiple stimulus sequences). (B, C) Schematics of stimulus sequences (composed of seven unique stimuli, one respective odor mix application at beginning and end) used for the individual datasets. Amino acid stimuli used for analysis across datasets are highlighted by the black frames. The lower right schematic illustrates the number of stimulus sequences and trials used for glomerular or MTC recordings.

(D) Multi-site Calcein AM dye injection into the MCL of the lateral OB (left panel). Colored circles represent differently colored dye reservoirs with overlapping areas. MTC dendritic staining depends on the position of the MTC soma and the ratio of the surrounding dyes. Example of multi-site Calcein dye injection with a lateral (red), intermedio-dorsal (blue) and medial (green) MTC population and their dendritic projection fields labeled (right panel).

(E) Fluorophore-coupled wheat germ agglutinin (WGA) application into the nostril of anesthetized tadpoles with subsequent active uptake by ORNs and anterograde, axonal transport to the OB.

(F) Single ORN electroporation with dextran-coupled fluorophores (magenta) in anesthetized tadpoles.

(G) Fluo-4 AM dye bulk injection into MCL of the olfactory system explant (yellow) including individual labeled ORN axon (magenta).

(H) CRE recombinase solution injection (blue) with subsequent bulk electroporation into the MOE of anesthetized, transgenic Brainbow tadpoles.

(I) Sparse cell electroporation of MTCs with fluorophore-coupled dextrans (red). ORNs and their axons express the fluorescent proteins Cerulean (cyan) or EYFP (yellow) induced by prior CRE mRNA injection/electroporation.

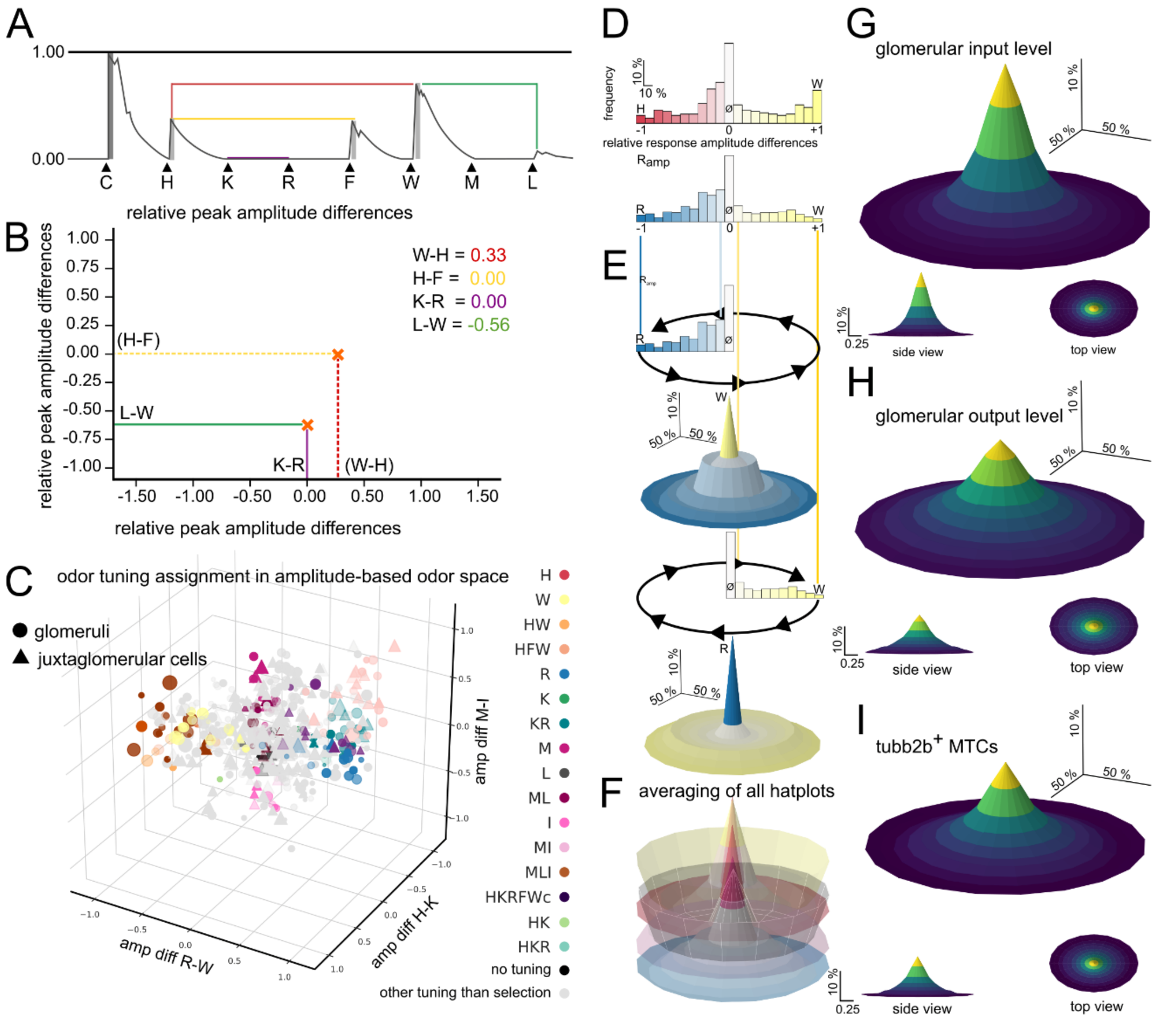

A
1.00
0.00
C
H
K
R
F
W
M
L
relative peak amplitude differences
B
W-H = 0.33
H-F = 0.00
K-R = 0.00
L-W = -0.56
(H-F)
L-W
K-R
(W-H)
relative peak amplitude differences
relative peak amplitude differences
C
odor tuning assignment in amplitude-based odor space
glomeruli
juxtaglomerular cells
amp diff R-W
amp diff H-K
amp diff M-I
H
W
HW
HFW
R
K
KR
M
L
ML
I
MI
MLI
HKRFWc
HK
HKR
no tuning
other tuning than selection
D
frequency
relative response amplitude differences
Ramp
E
F
averaging of all hatplots
G
glomerular input level
side view
top view
H
glomerular output level
side view
top view
I
tubb2b+ MTCs
side view
top view

Figure S2. Overall tuning broadness to single amino acids is narrowed from the input to the glomerular output and MTC level, Related to Figure 1

(A, B) Schematic of amplitude differences calculation for individual fluorescent time traces and use of amplitude differences as axes for 2D/3D odor space representation. Position of responsive region (orange cross) in 2D odor space using two pairings of response peak amplitude differences (K-R, magenta and L-W, orange or W-H, red and H-F, yellow).

(C) 3D odor space representation of individual glomeruli and JGCs of the output level defined by the three chosen amplitude difference axes R-W, H-K, and M-I (glomeruli: colored spheres; JGCs: colored triangles; diameter scaled to glomerular cross-sectional area). Colors assigned to individual (dominant) odor tunings see legend or Figure 2.

(D) Distribution of all glomerular regions (output level) according to their amplitude differences to two amino acid stimuli (H: red and W: yellow bars upper panel or R: blue, W: yellow lower panel).

(E) 3D hat-shaped representation of amplitude difference distributions ranging from 0 to -1 (blue shades) or 0 to +1 (yellow shades), hat tip color indicates the stimulus used for amplitude difference calculation.

(F) Schematic of averaging all possible 3D representations of amplitude differences distribution.

(G, H, I) Averaged 3D representations of amplitude difference distributions of the glomerular input (H), glomerular output (I), and *tubb2b*$^{+}$ MTC level (J). The individual concentric rings of the 'hats' represent frequency bins of response amplitude differences. The frequency values are plotted along the z-axis and color-coded (dark blue to lime).

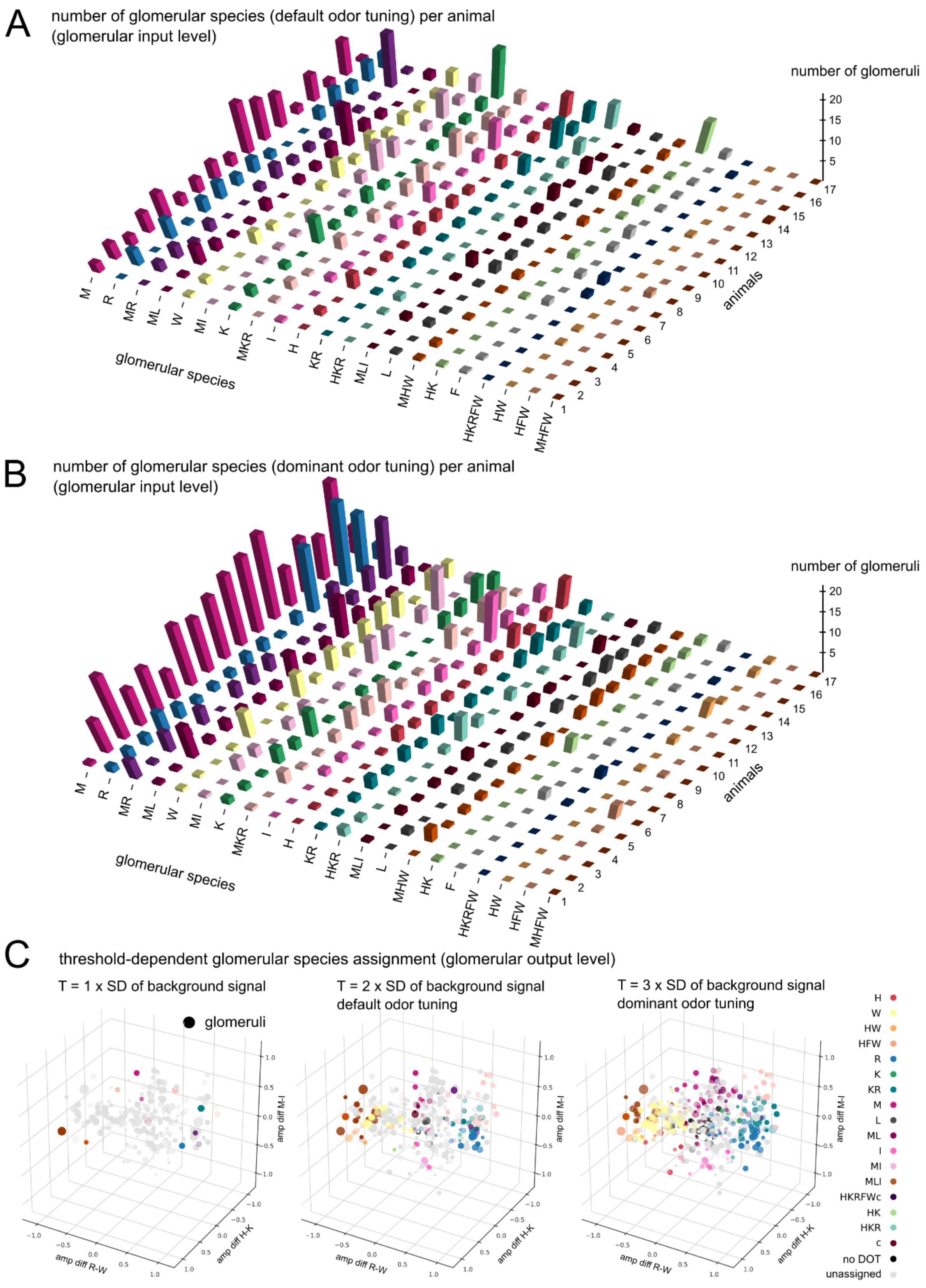
A
number of glomerular species (default odor tuning) per animal
(glomerular input level)
number of glomeruli
glomerular species
animals
M
R
MR
ML
W
MI
K
MKR
I
H
KR
HKR
MLI
L
MHW
HK
F
HKRFW
HW
HFW
MHFW
B
number of glomerular species (dominant odor tuning) per animal
(glomerular input level)
number of glomeruli
glomerular species
animals
C
threshold-dependent glomerular species assignment (glomerular output level)
T = 1 x SD of background signal
glomeruli
T = 2 x SD of background signal
default odor tuning
T = 3 x SD of background signal
dominant odor tuning
amp diff R-W
amp diff H-K
amp diff M-I
H
W
HW
HFW
R
K
KR
M
L
ML
I
MI
MLI
HKRFWc
HK
HKR
c
no DOT
unassigned

Figure S3. Lack of stereotypy in glomerular species numbers on the glomerular input level, Related to Figure 2

(A, B) 3D bar plots of numbers of glomerular species between the animals according to their default or dominant odor tuning based categorization. Numbers of glomerular species (input level, z-axis) of 21 frequent (dominant) odor tunings (x-axis) plotted for each animal (y-axis).

(C) Amplitude difference-based 3D odor space representation of glomeruli of the output level (spheres, x, y, z position in space: details see methods section and Figure S1A) with an additional assignment of odor tuning (colors) depending on the response threshold used (1x, 2x, or 3x SD of baseline/background fluorescence signal).

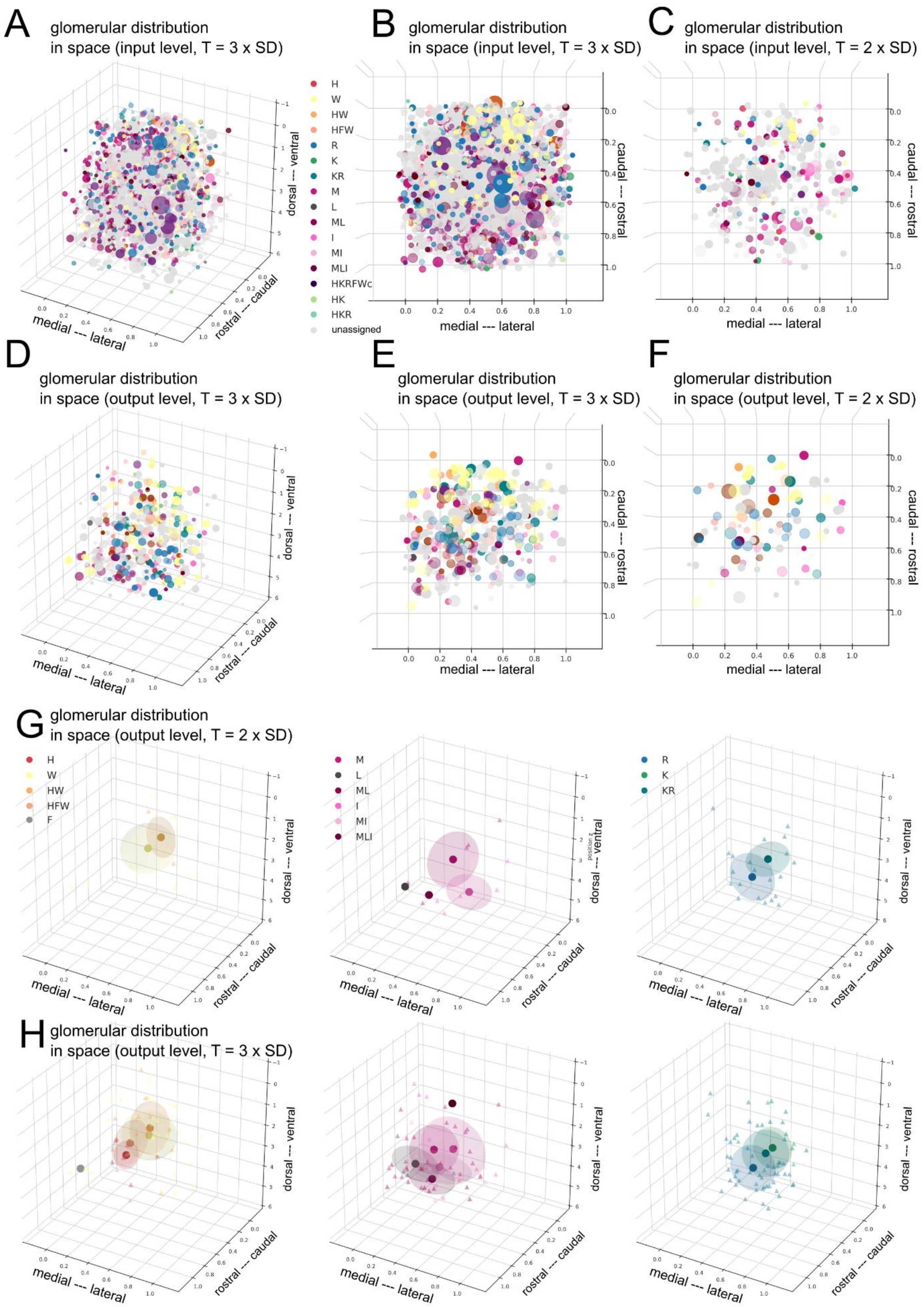
A glomerular distribution in space (input level, T = 3 x SD)
B glomerular distribution in space (input level, T = 3 x SD)
C glomerular distribution in space (input level, T = 2 x SD)
D glomerular distribution in space (output level, T = 3 x SD)
E glomerular distribution in space (output level, T = 3 x SD)
F glomerular distribution in space (output level, T = 2 x SD)
G glomerular distribution in space (output level, T = 2 x SD)
H glomerular distribution in space (output level, T = 3 x SD)
H
W
HW
HFW
R
K
KR
M
L
ML
I
MI
MLI
HKRFWc
HK
HKR
unassigned
F
medial --- lateral
rostral --- caudal
dorsal --- ventral
caudal --- rostral

Figure S4. Overlapping but differential positioning of glomeruli with similarity in ligand specificity on the glomerular level, Related to Figure 3

(A–F) Positioning of glomeruli pooled from all animals on the glomerular input (A, B, C; n=17) and output level (D, E, F; n=10). A, D) 3D distribution of glomeruli (default / dominant odor tunings assigned as colors) along the medio-lateral, caudo-rostral, and ventro-dorsal axes. Distribution along the medio-lateral and dorsal axes of glomeruli of dominant (B, E) or default odor tuning (C, F).

(G, H) 3D distribution of individual glomerular species (glomerular output level) grouped by their overarching selectivity to single or groups of structurally similar amino acids (left panels: aromatic amino acids H, F, W; middle panels; long-chain neutral amino acids M, L, I; right panels; basic amino acids K and R) along the medio-lateral, rostro-caudal and dorso-ventral axes according to their default (G) or dominant odor tuning (H). Positional variability of the individual glomerular species plotted as an ellipse in the dimensions of the standard deviation from the centroid of the data points (darker shaded circle in respective color). T, threshold; SD, standard deviation. Default/dominant odor tunings are represented by individual colors and combinations of their single letter codes.

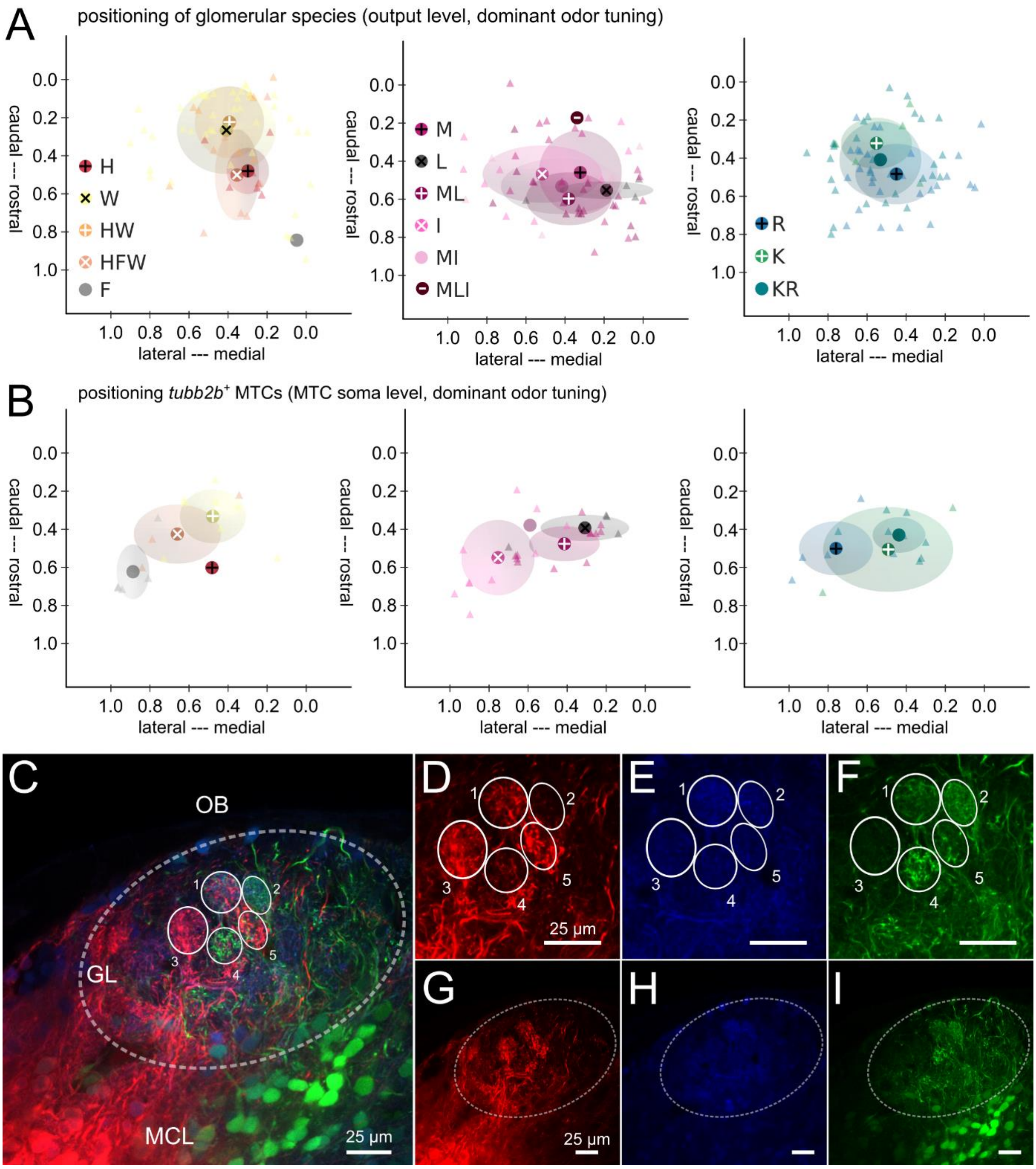
A
positioning of glomerular species (output level, dominant odor tuning)
caudal --- rostral
lateral --- medial
H
W
HW
HFW
F
M
L
ML
I
MI
MLI
R
K
KR
B
positioning tubb2b+ MTCs (MTC soma level, dominant odor tuning)
C
OB
GL
MCL
25 µm
D
E
F
G
H
I
25 µm
25 µm

Figure S5. MTC tufted dendrites innervate distinct glomerular units among the seemingly unparcellated lateral cluster, Related to Figure 3

(A, B) 2D projection of positioning of output level glomerular species (A) and *tubb2b*$^{+}$ MTC somata (B) along the medio-lateral and caudo-rostral axes, grouped by dominant odor tuning. Positional variability of the individual glomerular species/MTC somata plotted as an ellipse in the dimensions of the standard deviation from the centroid of the data points (darker shaded circle).

(C) Example of differential staining of MTCs using Calcein Red in the lateral (red), Calcein Violet in the intermedio-ventral (blue), and Calcein Green in the medial MCL (green).

(D, E, F) Section of the lateral cluster with differential innervation of individual glomeruli (white numbered ellipses) by differently colored postsynaptic dendritic arborizations (C, composite image; D, Calcein Red, E, Calcein Violet; F, Calcein Green).

(G, H, I) Projections from the lateral (red), intermediate (blue), and medial (green) MCL were biased to the lateral, intermediate, and medial glomerular layer, respectively.

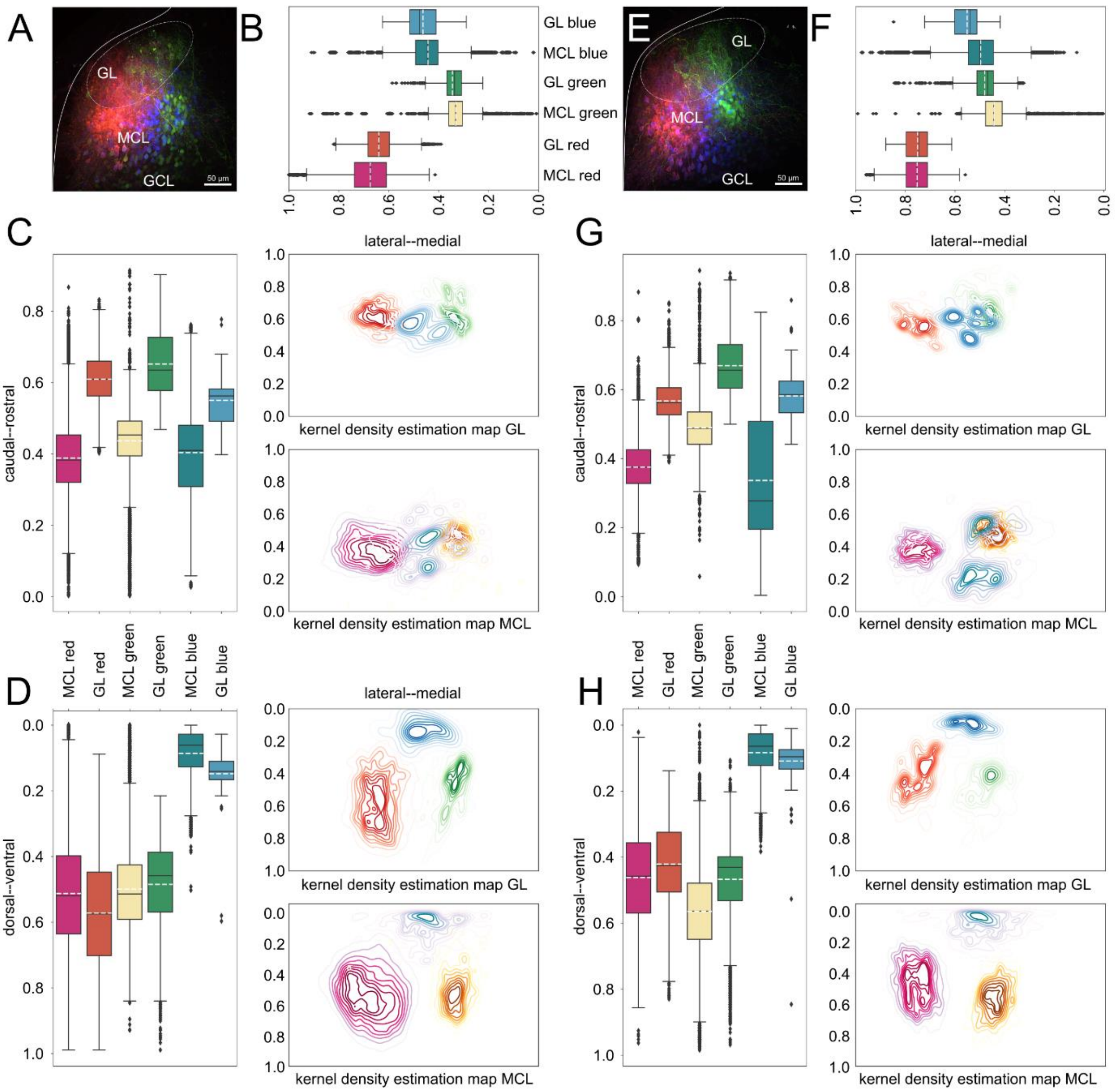

Figure S6. MTC somata and their primary dendritic projections are arranged in a coarse topological manner in the lateral OB, Related to Figure 3

Comparison of somatic positions of defined MTC subsets to their primary dendritic projection fields across the lateral cluster (in two animals, A-D and E-G). A, E) Maximum projection image of multi-site Calcein AM dye injection (see methods) into the mitral cell layer (MCL) of the lateral OB after 1 h of incubation. Differentially colored primary dendritic projections of neurons of the MCL are visible in the glomerular layer (GL; dashed line; blue, green, red). B, F) Topologically linked distributions of ‘red’, ‘blue’ and ‘green’ pixels (color ratio categorization and thresholding see methods) between the GL (blue, green & red) and the MCL (teal, yellow & magenta) along the lateral to medial axis. C & D, G & H) Kernel density estimation maps of ‘red’, ‘blue’ and ‘green’ pixels of the GL (blue, green & red; upper panel) and the MCL (teal, yellow & magenta; lower panel) as combination of their medial to lateral and C, D) caudal to rostral or D, H) dorsal to ventral distributions (see boxplots on the left for distribution along the latter axes only). Mean, dashed white line; median: black line; box outlines 1st and 3rd quartiles.

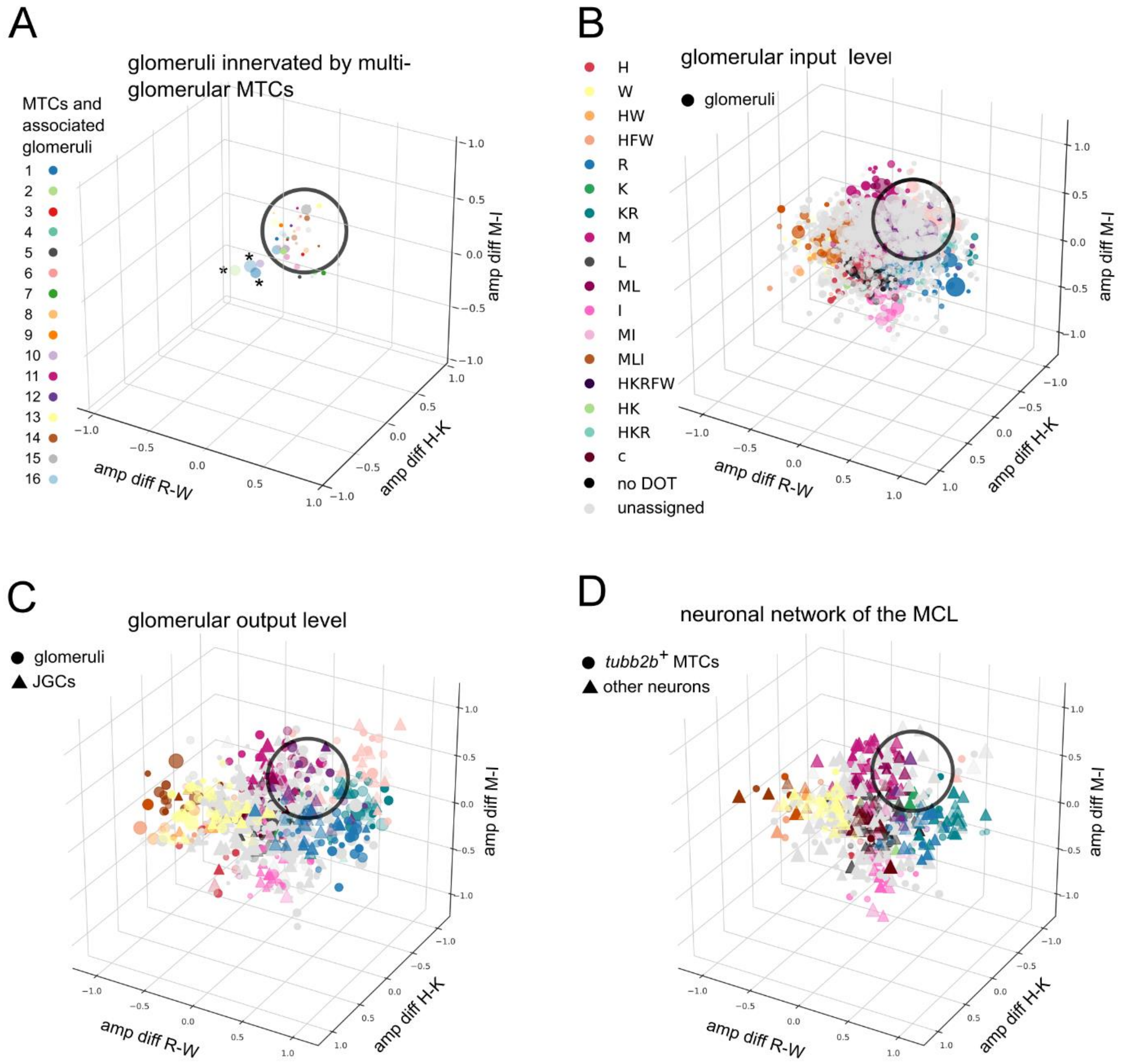

Figure S7. Response amplitude difference-based 3D odor space representations of the multiple levels of OB odor processing, Related to Figure 7

(A) Focal cluster of glomeruli innervated by multi-glomerular MTC (grey circle) in 3D odor space representation. Colors represent association to individual MTCs (left legend) and glomeruli of 'deviant MTCs' 1 and 2 (Figure 6) are labeled with asterisks.

(B) Odor space representation of glomerular input level dataset. Glomeruli are additionally assigned a color of dominant odor tuning (see left legend). Highlighted odor space section densely packed with glomeruli of broader amino acid tuning (grey color: unassigned dominant odor tuning).

(C) More widely spread distribution of glomeruli (circles; JGCs: triangles) of the output level in 3D odor space (see also Figure 1 and S2, higher fraction of selectively tuned glomeruli). Densities of glomeruli with similar tuning/odor space clustering as glomeruli innervated by multi-glomerular MTCs (grey circles) are lower on glomerular output and (D) MTC soma level (circles: *tubb2b*$^{+}$ MTCs, circles: all neurons in MCL proximity).

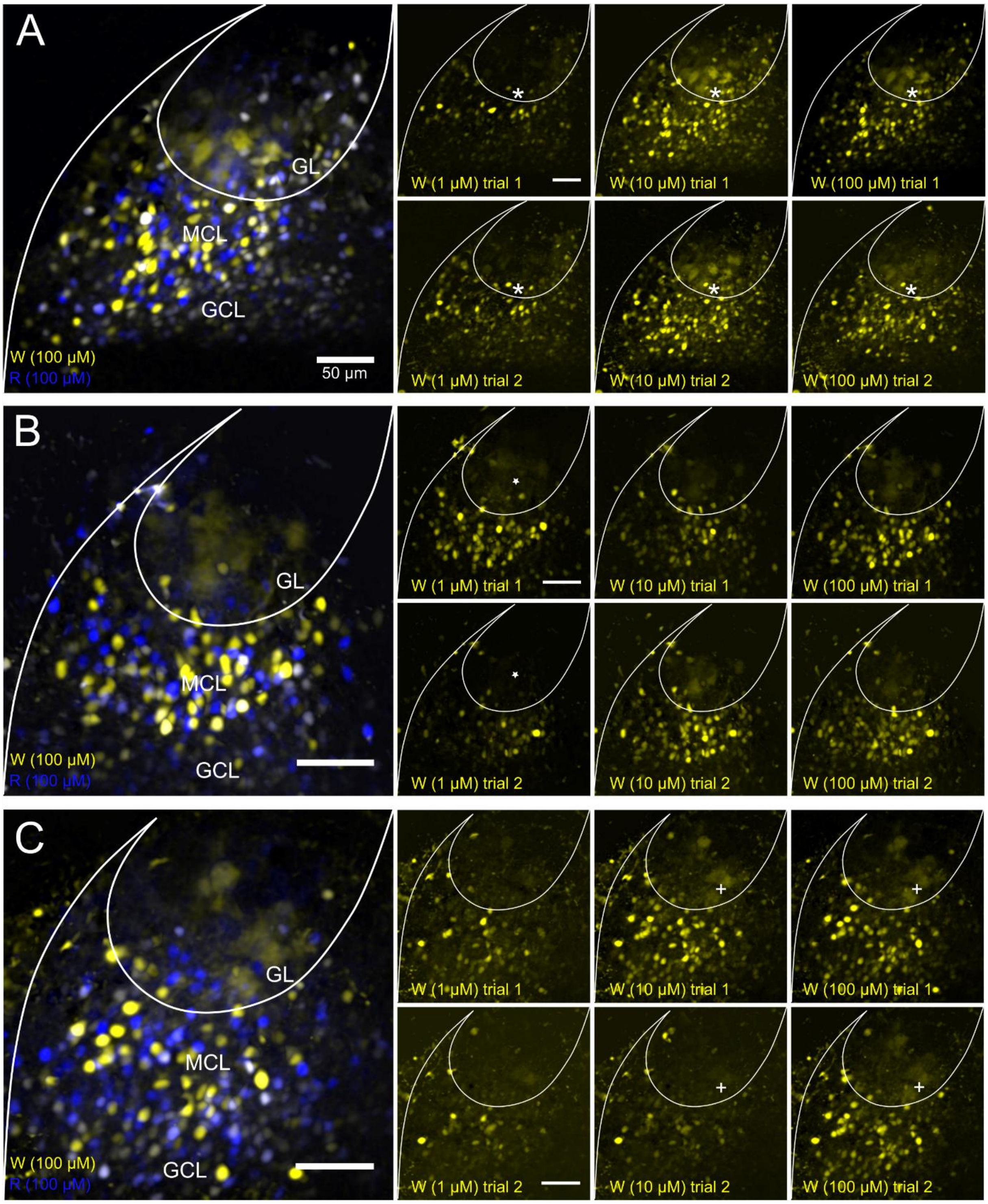
A
GL
MCL
GCL
W (100 μM)
R (100 μM)
50 μm
W (1 μM) trial 1
W (10 μM) trial 1
W (100 μM) trial 1
W (1 μM) trial 2
W (10 μM) trial 2
W (100 μM) trial 2
B
GL
MCL
GCL
W (100 μM)
R (100 μM)
W (1 μM) trial 1
W (10 μM) trial 1
W (100 μM) trial 1
W (1 μM) trial 2
W (10 μM) trial 2
W (100 μM) trial 2
C
GL
MCL
GCL
W (100 μM)
R (100 μM)
W (1 μM) trial 1
W (10 μM) trial 1
W (100 μM) trial 1
W (1 μM) trial 2
W (10 μM) trial 2
W (100 μM) trial 2

Figure S8. Maximally activatable glomerular ensembles to single amino acids stabilize at higher odor concentrations, Related to Figure 1

Intensity difference maps (maximum projections) of three different lateral OBs to R (blue) or W (yellow). Left, big panels: composite image of odor representations (intensity difference maps) to W and R at 100 µM stimulus concentration. Right small panels: Intensity difference-based odor maps to W applied at 1, 10 and 100 µm respectively (left to right) performed in two trials (upper vs. lower row).

(A) animal with lack of glomerular odor representation to W at 1 µM concentration vs stable representations in the glomerular layer from 10 µM on (white asterisks).

(B, C) Higher inconsistencies in maximally activatable glomerular ensemble by W at 1 µM (white stars) and even 10 µM concentrations (white crosses).
